# Estimation of the incidence of animal rabies in Punjab, India

**DOI:** 10.1371/journal.pone.0222198

**Published:** 2019-09-09

**Authors:** Gurlal S. Gill, Balbir B. Singh, Navneet K. Dhand, Rabinder S. Aulakh, Bhupinder S. Sandhu, Michael P. Ward, Victoria J. Brookes

**Affiliations:** 1 Guru Angad Dev Veterinary and Animal Sciences University (GADVASU), Ludhiana, Punjab, India; 2 Punjab Agricultural University (PAU), Ludhiana, Punjab, India; 3 Sydney School of Veterinary Science, Faculty of Science, The University of Sydney, Camden, NSW, Australia; 4 School of Animal and Veterinary Sciences, Faculty of Science, Charles Sturt University, Wagga Wagga, NSW, Australia; University of Minnesota, UNITED STATES

## Abstract

**Background:**

Rabies is a devastating zoonotic disease of mammals that causes encephalitis and death. It is endemic in India, with an estimated annual 20,000 human deaths (one-third of the global rabies burden). The magnitude of animal rabies incidence is unknown.

**Methods:**

In four sub-districts of Punjab, India, we monitored canine and livestock populations from August 15, 2016 to August 14, 2017. Demographic, clinical and rabies diagnostic laboratory (RDL) data were collected from suspected cases of rabies. The annual incidence rate / 10,000 animal years at risk (95% CI) in each sub-district was estimated for each species.

**Results:**

During 2016–2017, a total of 41 suspected rabies cases were detected in the four selected sub-districts in Punjab. Laboratory confirmed rabies (LCR) incidence was 2.03/10,000 dog years (0.69, 5.96) and 2.71/10,000 dog years (1.14, 6.43) in stray and pet dogs, respectively. The LCR incidence in farmed buffalo and cattle was 0.19/10,000 buffalo years (0.07, 0.57) and 0.23/10,000 cattle years (0.06, 0.88), respectively. The LCR incidence amongst equine was 4.28/10,000 equine years (0.48, 38.10). Stray cattle rabies incidence in the selected sub-districts was 9.49/10,000 cattle years (3.51, 25.67). If similar enhanced surveillance for rabies was conducted state-wide, we estimate that 98 (34–294) buffalo, 18 (2–156) equine, 56 (15–214) farmed cattle, 96 (35–259) stray cattle, 128 (54–303) pet dogs and 62 (21–182) stray dogs would be expected to be confirmed with rabies in Punjab annually.

**Conclusion:**

These results indicate that rabies incidence in animals, particularly in dogs and stray cattle, is much higher than previously suspected. We recommend that statewide enhanced disease surveillance should be conducted to obtain more accurate estimates of rabies incidence in Punjab to facilitate better control of this important disease.

## Background

Rabies is a devastating and fatal zoonotic disease of mammals [1]. Rabies virus is primarily transmitted through the bite of a rabid animal. Domestic dogs are rabies virus reservoirs in African and Asian countries [2, 3]. Although the first vaccine against rabies was developed by Louis Pasteur in 1885 [4], rabies remains a neglected disease in humans and animals, particularly in poor and marginalized populations in low-resource settings, where it is often endemic [5]. It is estimated that canine rabies poses a threat to more than 3.3 billion people worldwide [4].

Rabies in dogs can present as ‘furious’ or ‘paralytic’ forms. Rabid cats have been reported to be more violent than rabid dogs [6, 7]. In contrast, the signs in bovine include excessive salivation, behavioral change, vocalization, and pharyngeal paresis [8]. The disease is untreatable and eventually causes death of affected animals.

Recent assessments suggest that worldwide, approximately 59,000 people die of rabies annually, mostly in Asia and Africa [9]. In India, the annual incidence of rabies has been estimated to be at least 2/100,000 people (20,000 deaths annually; one-third of the annual global rabies burden [10, 11]). Rabies is not a notifiable disease in India and there is no organized surveillance system for either human or animal cases.

Rabies also causes a huge health and economic impact in human and animal populations. It has been estimated that globally, canine rabies caused approximately 3.7 million disability-adjusted life years (DALYs; 95% CIs: 1.6−10.4 million) [10, 12]. Further, the annual cost of livestock losses as a result of rabies has been estimated to be USD 12.3 million in Africa and Asia [12].

Stray dogs are responsible for most of the human and animal bite cases in India [13, 14] and dogs are considered an important reservoir host for rabies [15], responsible for > 97% of human rabies deaths [2]. The elimination of canine rabies, particularly in stray dogs, is a goal for rabies control in India. Factors such as poor dog population management and low standards of dog care (including infrequent veterinary consultation) are considered responsible for high endemicity of canine rabies in India [13].

In India, information is not available about the incidence of rabies in animal populations. Recent studies using passive rabies diagnostic laboratory (RDL) data have revealed that rabies is an endemic disease in Punjab [16]. However, passive reports often underestimate disease incidence. There is a lack of accurate quantitative information about the status of rabies in animal populations, especially in stray animal populations (which play an important role in endemicity of the disease; [16]). Therefore, the current study was planned to estimate the annual incidence of rabies in canine and livestock populations and to compare incidence across species in Punjab, India, based on laboratory submissions. We also aimed to describe the clinical history and signs associated with laboratory confirmed cases. This information will be used to inform policy to develop control programs for the eradication of rabies in domestic animal populations in Punjab, India.

## Methods

### Ethics statement

The study protocol was approved by the Institutional Ethics Committee, Dayanand Medical College and Hospital, Ludhiana, Punjab (approval DMCH/R&D/2017/482). Participants were provided with a Participant Information Statement explaining the purpose of the study, and written consent was obtained from all participants before data for the study were collected. The animal care and use protocol adhered to the Committee for the Purpose of Control and Supervision of Experiments on Animals, Government of India. Note that no experimental animals/procedures were involved, animals were not subjected to euthanasia for the purposes of this study, and the on-site available information was collected from veterinary personnel/farmers.

### Study area

The study was conducted in Punjab State, India. Punjab is an agricultural state located in north-west India, with populations of approximately 27.74 million humans and 0.47 million domestic dogs [17, 18]. The state has a total livestock population of 8.1 million including 2.4 million cattle, 5.2 million buffalo, 0.45 million small ruminants and 0.04 million equines [18]. Punjab has 22 districts and 81 sub-districts covering an area of 50,362 km^2^ (http://punjab.gov.in/know-punjab). The state has a veterinary university (Guru Angad Dev Veterinary & Animal Sciences University; GADVASU) and the rabies diagnostic laboratory (RDL) in this university is the only diagnostic facility for animal rabies in Punjab.

### Sub-district selection

**Fig 1:**
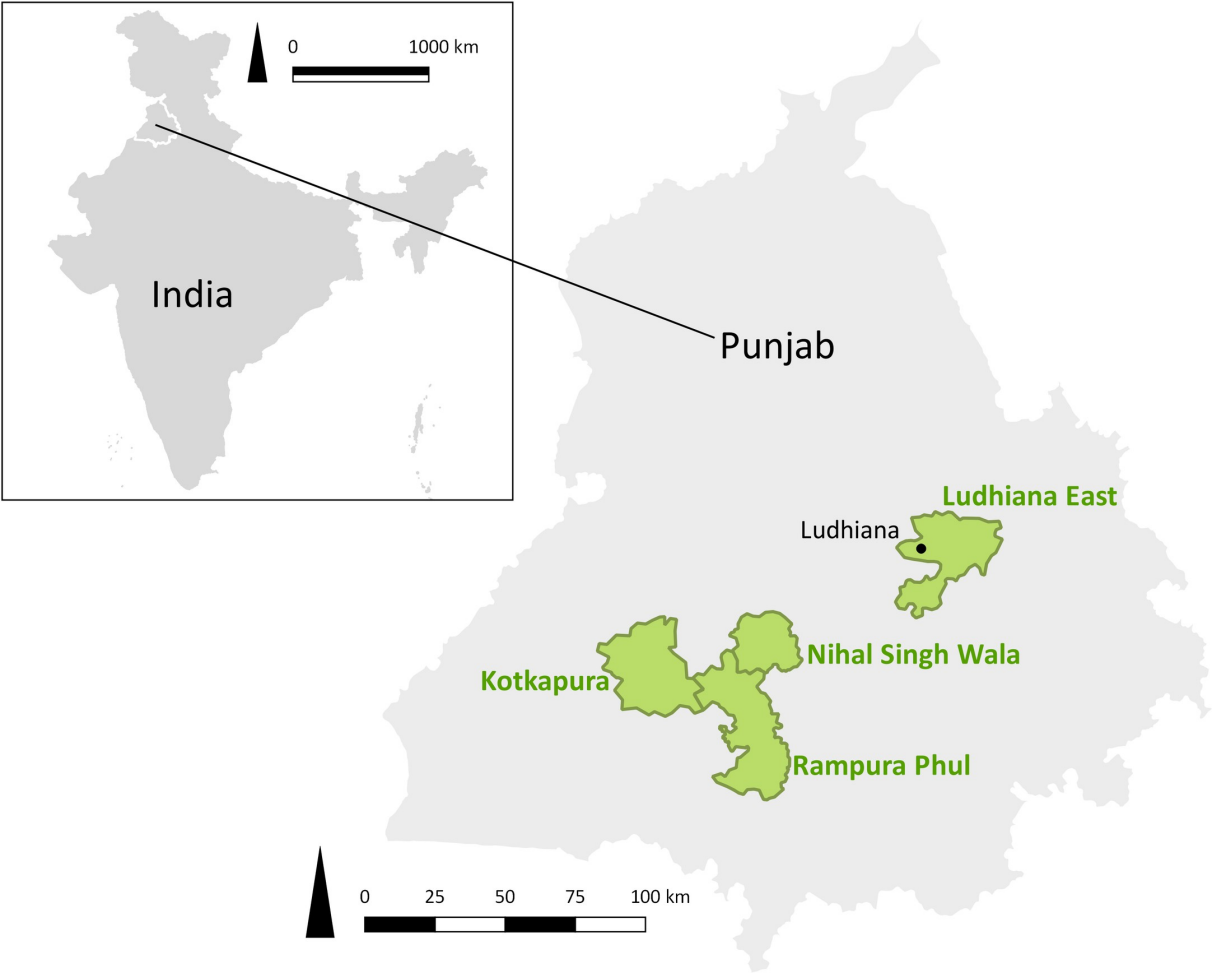
Location of sub-districts (Ludhiana East 30.931423, 75.899679; Kotkapura 30.582452, 74.819616; Nihal Singh Wala 30.591006, 75.278728; Phull 30.323492, 75.241006) selected in a study of rabies incidence in animals in Punjab, India, between August 15, 2016 and August 14, 2017. QGIS 3.6.0 was used to create the figure (qgis.org). The shapefiles are publicly available from diva-gis.org (India and Punjab) or created by the authors (districts).

Information derived from rabies case records from the GADVASU RDL was used to select the sub-districts for the current study [16]. Based on the animal rabies cases previously reported from the sub-districts in Punjab, the sub-districts were divided into four groups: no rabies cases reported (n = 27), 1–6 rabies case reported (n = 41), 7–12 rabies case reported (n = 8) and those reporting > 12 rabies cases (n = 5) during the period 2004–2014 (S1 Table). A sub-district from each group was selected using a random number generator. The selected sub-districts were Kotkapura (0 cases), Phull (4 Cases), Nihal Singh Wala (9 cases) and Ludhiana East (46 cases) (Fig 1).

### Population sizes

The sub-district level livestock, pet and stray animal population data (19^th^ Livestock Census) were provided by the respective district offices of the Deputy Director, Department of Animal Husbandry, Punjab (S2 Table). State and national level data (S2 Table) were obtained from official data sources [18, 19].

Population sizes for stray dogs and cattle were not available for the sub-districts Nihal Singh Wala and Phull, and the population size for equines was not available for the sub-district Nihal Singh Wala. Therefore, we imputed these missing values using a random forest algorithm from the missForest package in R [20, 21], taking into account the sub-district level population data available for other domestic and stray animal populations in the sub-district with missing data and in the other sub-districts in the study. We assumed that the data were “missing at random.” The normalized root mean squared error was 0.009 following imputation.

### Sub-district monitoring

The livestock and canine populations in the selected sub-districts were prospectively monitored for a period of one year from August 15, 2016 to August 14, 2017. These sub-districts contained both villages and wards, the smallest organizational levels in rural and urban areas, respectively. Lists of veterinary doctors and veterinary pharmacists (designation used for veterinary nurses; also called veterinary inspectors) serving in the selected sub-districts were obtained from the respective offices of the Deputy Director, Department of Animal Husbandry, Punjab. The veterinary doctors and pharmacists working in the selected sub-districts were requested to report any suspected rabies case encountered in their area to the researchers using a questionnaire designed for this purpose (see below). Researchers contacted veterinary doctors and pharmacists by telephone regularly throughout the period of the study to receive updates about new cases in their area. Information about the study and researcher contact information was pasted (as posters) on public places in all villages and wards. Livestock farmers and the general public were asked to report any suspected rabies case either to the department veterinarians, veterinary pharmacists or directly to the School of Public Health and Zoonoses, GADVASU.

### Questionnaire design and information collection

A detailed questionnaire was designed for the collection of demographic data, vaccination status, geographic co-ordinates and the timeline of all suspected cases (S1 and S2 Appendix). For pet and domestic animals, the information was collected from the owner. For stray animals, available data at the site of observation were collected. The identifying marks of stray animals were recorded, when suspected live animals were encountered, at the time of clinical evaluation to ensure identification between animal carcasses and the suspected rabid animal. The exposure history of humans and healthy animals from suspected rabies case were also recorded using a pre-designed questionnaire.

### Clinical evaluation

Suspected cases of animal rabies reported from the selected sub-districts were clinically evaluated by the local veterinary doctor or veterinary pharmacist and were later evaluated by the lead author (who is also a veterinarian). The disease history and the clinical information reported by the veterinarian or pharmacist were recorded. Occupational exposures and exposures to other humans or animals were also recorded.

### Laboratory testing

Brain samples from suspected rabies-infected animals were collected post-mortem. The person involved in the collection of samples received pre-exposure anti-rabies vaccination and wore personal protective equipment sufficient to prevent rabies exposure during the collection of the samples. Rabies was diagnosed via the direct fluorescent antibody technique (dFAT; [22]) in the RDL at GADVASU. Laboratory data were recorded and compiled with the relevant case history.

### Data handling and statistical analysis

Data were entered into an Excel spreadsheet (Microsoft Office Excel 2007) and checked for transcribing errors and consistency (for example, date format). All statistical analyses were conducted in the SAS statistical program ver. 9.4 (2002–2012 by SAS Institute Inc., Cary, NC, USA.) and the graphs were created using R statistical program (R statistical package version 3.4.0, R Development Core Team [2015], http://www.r-project.org) unless indicated otherwise.

Descriptive statistics and frequency distributions were generated for clinical signs, exposure histories and types of exposure to other susceptible animals and humans for each sub-district, and species for all laboratory confirmed rabies cases.

Summary statistics for the number of rabies cases (both laboratory-confirmed and clinically suspected) were calculated by species and sub-district. Poisson regression models were then fitted using the GLIMMIX procedure in SAS by including the number of clinically suspected / laboratory confirmed cases as an outcome, species as a fixed effect, sub-district as a random effect and (a log of) the population of each species in each sub-district as an offset. Parameter estimates (95% CI) were exponentiated to calculate incidence rate ratios (95% CI). The annual incidence / 10,000 animal years at risk (95% CI) for each species was estimated by calculating predicted means after applying the inverse-link transformation and then multiplying by 10,000. The goodness-of-fit chi-squared test was used to evaluate the fit of the model. Studentized residuals were plotted to evaluate the assumptions of the model.

The annual incidence / 10,000 animal years at risk (95% CI) was used to estimate the number of rabies cases that would be reported for each species if the surveillance methods in this study were implemented state-wide.

## Results

During 2016–2017, a total of 41 suspected rabies cases were reported by veterinary doctors (n = 25), veterinary pharmacists (n = 12) and farmers (n = 4) in the selected sub-districts (Table 1). Of these 41 suspected cases, 33 whole brains were submitted to the RDL at GADVASU of which 30 (91%) were confirmed infected with rabies virus. Eight samples from the suspected cases could not be submitted to the RDL; these were considered as suspect cases by the veterinary doctors (6) and veterinary pharmacists (2) based on clinical signs.

**Table 1:** Suspected rabies cases reported by veterinary doctors, veterinary pharmacists and dairy farmers (preliminary judgement of ‘positive’ and ‘not sure’ based on clinical signs) and subsequent laboratory confirmation of rabies cases reported from selected sub-districts in Punjab, India, from August 15, 2016 to August 14, 2017.

	Veterinary Doctor (Diagnosis based on clinical signs)		Veterinary Pharmacist (Diagnosis based on clinical signs)		Collected from Dairy farmer (Diagnosis based on clinical signs)		Total
	Positive	Not sure	Positive	Not sure	Positive	Not sure	
GADVAS* **University Rabies Laboratory Result**							
**FAT (+)**	16	2	8	1	3	0	30
**FAT (-)**	0	1	1	0	1	0	3
**Not tested**	6	0	2	0	0	0	8
**Total**	22	3	11	1	4	0	41

*Guru Angad Dev Veterinary and Animal Sciences

**Fig 2:**
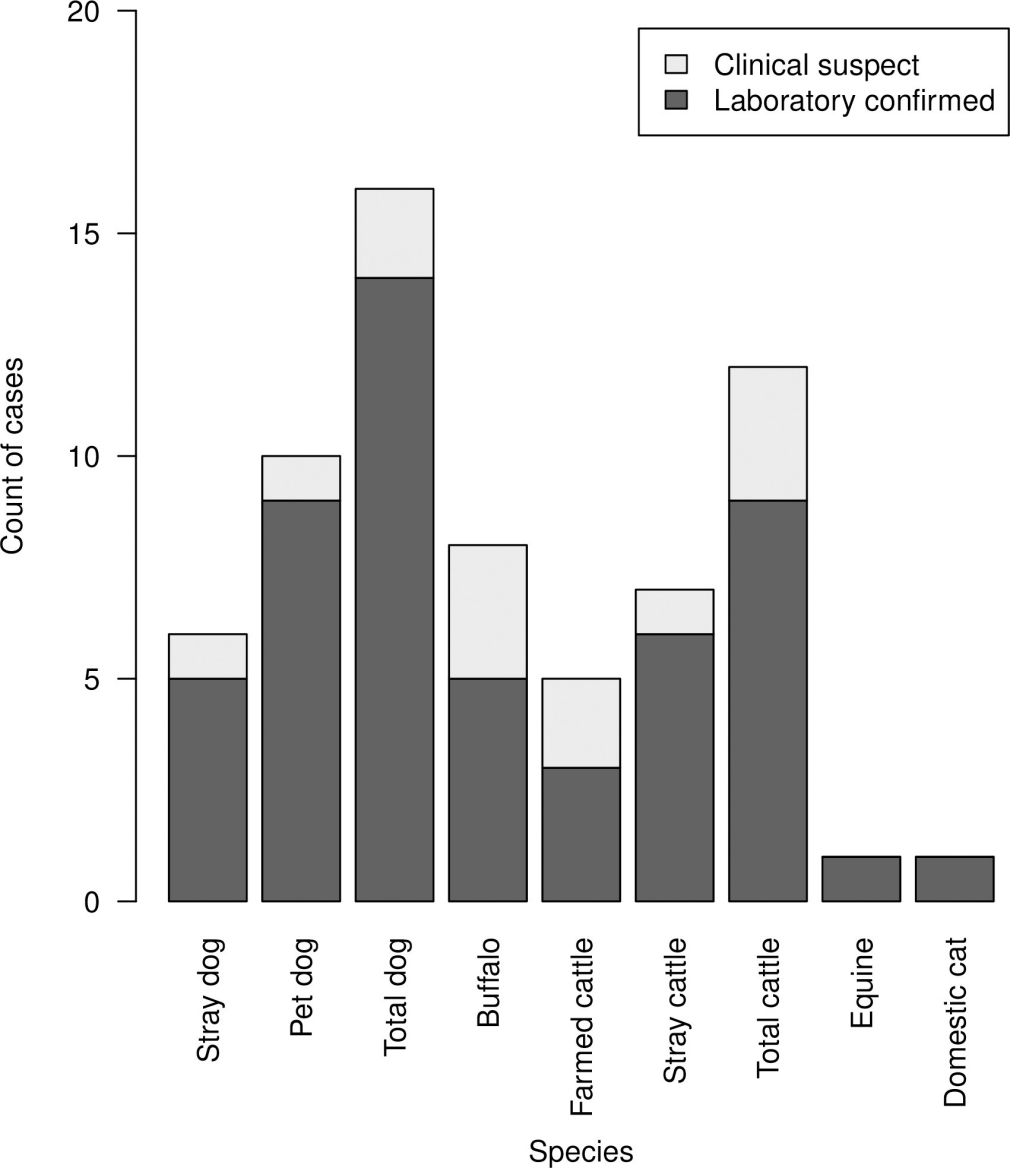
Number of clinically suspected and laboratory confirmed rabies cases reported in a study of rabies incidence in animals in selected sub-districts of Punjab, India, between August 15, 2016 and August 14, 2017.

Laboratory confirmed (n = 30) and clinically suspected (n = 8, cases that could not be laboratory confirmed) rabies cases (hereafter referred to as ‘rabies cases’) were recorded in the canine (n = 16), cattle (n = 12) and buffalo (n = 8) populations in all sub-districts (Table 2; Fig 2). Additionally, one feline and one equine (mule) rabies case was detected from Ludhiana East and Phull, respectively. No rabies case was recorded in small ruminants. Of confirmed rabies cases in cattle and buffalo, 58.3% and 87.5% were recorded in female cattle and female buffalo, respectively. A total of 56.2% dog cases were recorded from male dogs.

**Table 2:** Number of rabies cases by species and sub-district in selected sub-districts in Punjab, India, from August 15, 2016 to August 14, 2017.

Rabies cases/Total animal population	Stray dogs	Pet dogs	Total dogs	Stray cattle	Farmed cattle	Total cattle	Buffalo	Equine	Feline
Sub-district 1 (Kotkapura)									
N_1_	0	1	1	2	0	2	2	0	0
N_2_	0	1	1	3	0	3	3	0	0
P	1201	4175	5376	928	19565	20493	28191	287	-
Sub-district 2 (Ludhiana East)									
N_1_	3	1	4	0	1	1	2	0	1
N_2_	3	1	4	0	2	2	2	0	1
P	11503	17526	29029	2258	51308	53566	107838	774	-
Sub-district 3 (Nihal Singh Wala)									
N_1_	1	2	3	0	1	1	0	0	0
N_2_	2	2	4	0	1	1	0	0	0
P	6249*	5899	10602	1487*	20360	21091	40080	576*	-
Sub-district 4 (Phull)									
N_1_	1	5	6	4	1	5	1	1	0
N_2_	1	6	7	4	2	6	3	1	0
P	6970*	7748	14489	1673*	36238	37841	81326	680	-
Sub-districts (Combined estimates)									
N_1_	5	9	14	6	3	9	5	5	1
N_2_	6	10	16	7	5	12	8	1	1
P	25923	35348	59496	6346	127471	132991	257435	1741	-

*calculation (see text)

N_1_: Number of cases (lab confirmed); N_2_: Number of cases (lab confirmed + clinically suspected); P: Total animal population

**Fig 3:**
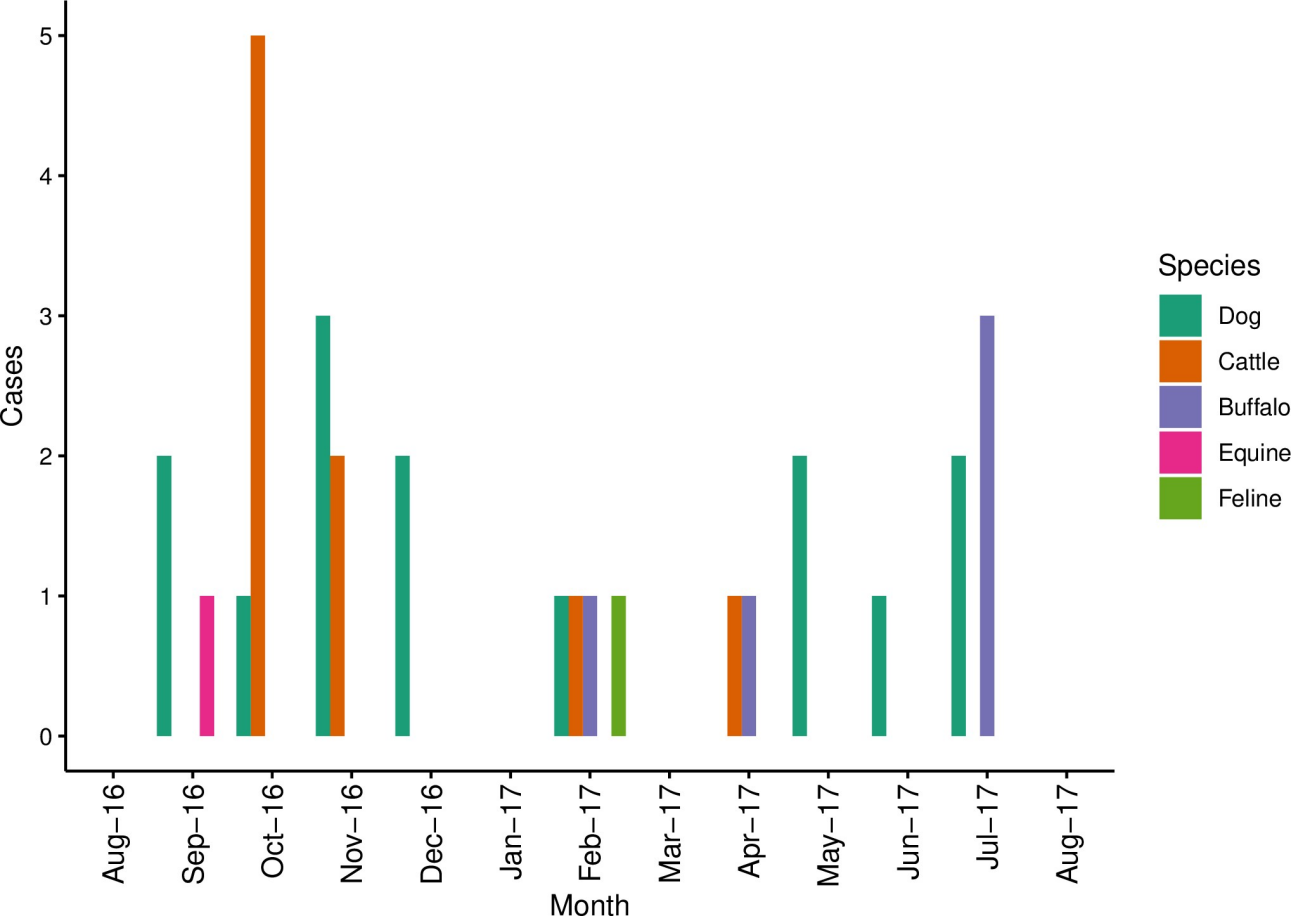
Monthly number of clinically suspected and laboratory confirmed rabies cases reported in a study of rabies incidence in animals in selected sub-districts of Punjab, India, between August 15, 2016 and August 14, 2017.

The rabies cases recorded in different months are presented in Fig 3.

Rabies cases in dogs were reported each month (range: 1−3 cases/month) from September to December 2016. In livestock species (cattle, buffalo and mule), sporadic rabies cases were reported, except during October 2016 when a peak of 5 rabies cases were reported in cattle.

### Annual incidence of rabies

During this study, suspected rabies cases were reported in cattle from all four sub-districts. The maximum numbers of cases were reported from Phull (n = 6) and minimum number of cases were reported from Nihal Singh Wala (n = 1). Most suspected rabies cases in stray dogs were from Ludhiana East (n = 3), with no cases from Kotkapura. In pet dogs, the maximum number of suspected rabies cases was reported from Phull (n = 6) and a single case was reported from both Kotkapura and Ludhiana East.

Rabies incidence in different species is presented in Table 3. Incidence was highest in stray cattle and lowest in farmed buffalo.

**Table 3:** Rabies incidence (per 10000 animal years at risk) by species in Punjab, India from August 15, 2016 to August 14, 2017.

Species	Rabies incidence (per 10,000 animal years at risk)					
	All cases			Laboratory confirmed		
	Mean	Lower CI	Upper CI	Mean	Lower CI	Upper CI
**Farmed buffalo**	0.31	0.12	0.80	0.19	0.07	0.57
**Equine**	4.24	0.47	38.63	4.28	0.48	38.10
**Farmed cattle**	0.39	0.13	1.18	0.23	0.06	0.88
**Pet dog**	3.05	1.26	7.41	2.71	1.14	6.43
**Stray cattle**	10.99	4.09	29.56	9.49	3.51	25.67
**Stray dog**	2.48	0.87	7.09	2.03	0.69	5.96

The parameter estimates and incidence rate ratios are presented in Table 4. The parameter estimates are the expected differences in log count of each group from the reference group (stray dog). The incidence of rabies in stray cattle was more than four times the incidence in stray dogs whereas the rabies incidence in farmed cattle and buffalo were significantly lower than that of stray dogs.

**Table 4:** Parameter estimates and rabies incidence rate ratios (IRR) by species in selected sub-districts in Punjab (India) from August 15, 2016 to August 14, 2017 (*reference level).

Outcome	Species	Estimate	SE	IRR	P-value
Clinically suspected cases	(Intercept)	-8.30	0.49	−	−
	Farmed buffalo	-2.09	0.54	0.12 (0.04, 0.39)	0.002
	Equine	0.54	1.08	1.71 (0.17, 17.14)	0.626
	Farmed cattle	-1.86	0.61	0.16 (0.04, 0.57)	0.008
	Pet dog	0.21	0.52	1.23 (0.41, 3.72)	0.695
	Stray cattle	1.49	0.56	4.43 (1.35, 14.58)	0.018
	Stray dog*	0.00	−	1.00	−
Laboratory confirmed cases	(Intercept)	-8.50	0.51	−	−
	Farmed buffalo	-2.35	0.63	0.10 (0.02, 0.37)	0.002
	Equine	0.75	1.10	2.11 (0.20, 21.79)	0.507
	Farmed cattle	-2.16	0.73	0.12 (0.02, 0.55)	0.010
	Pet dog	0.29	0.56	1.34 (0.41, 4.40)	0.612
	Stray cattle	1.54	0.61	4.67 (1.28, 17.06)	0.023
	Stray dog*	0.00	−	1.00	−

For RDL confirmed cases, we estimated the following annual rabies case numbers (95% CI) in Punjab: 98 (34–294) buffalo, 18 (2–156) equine, 56 (15–214) farmed cattle, 96 (35–259) stray cattle, 128 (54–303) pet dogs, 62 (21–182) stray dogs. The case numbers after including suspected cases would be: 160 (62–413) buffalo, 17 (2–158) equine, 95 (32–286) farmed cattle, 111 (41–299) stray cattle, 144 (59–349) pet dog and 76 (27–217) stray dogs.

### Clinical signs associated with canine and bovine rabies

**Fig 4:**
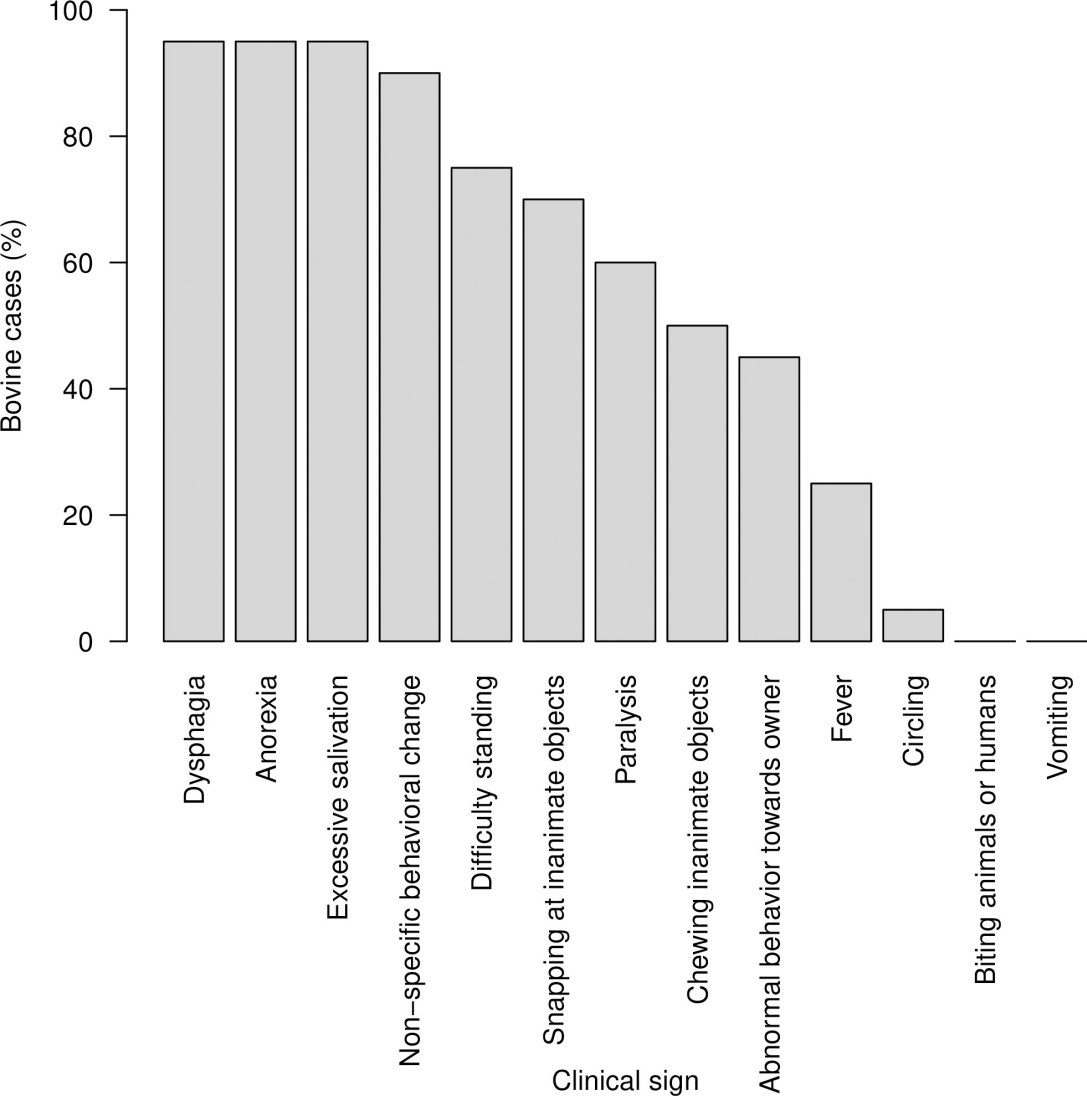
Clinical signs observed in 16 dogs with clinically suspected or laboratory confirmed rabies, reported in a study of rabies incidence in animals in selected subdistricts of Punjab, India, between August 15, 2016 and August 14, 2017.

**Fig 5:**
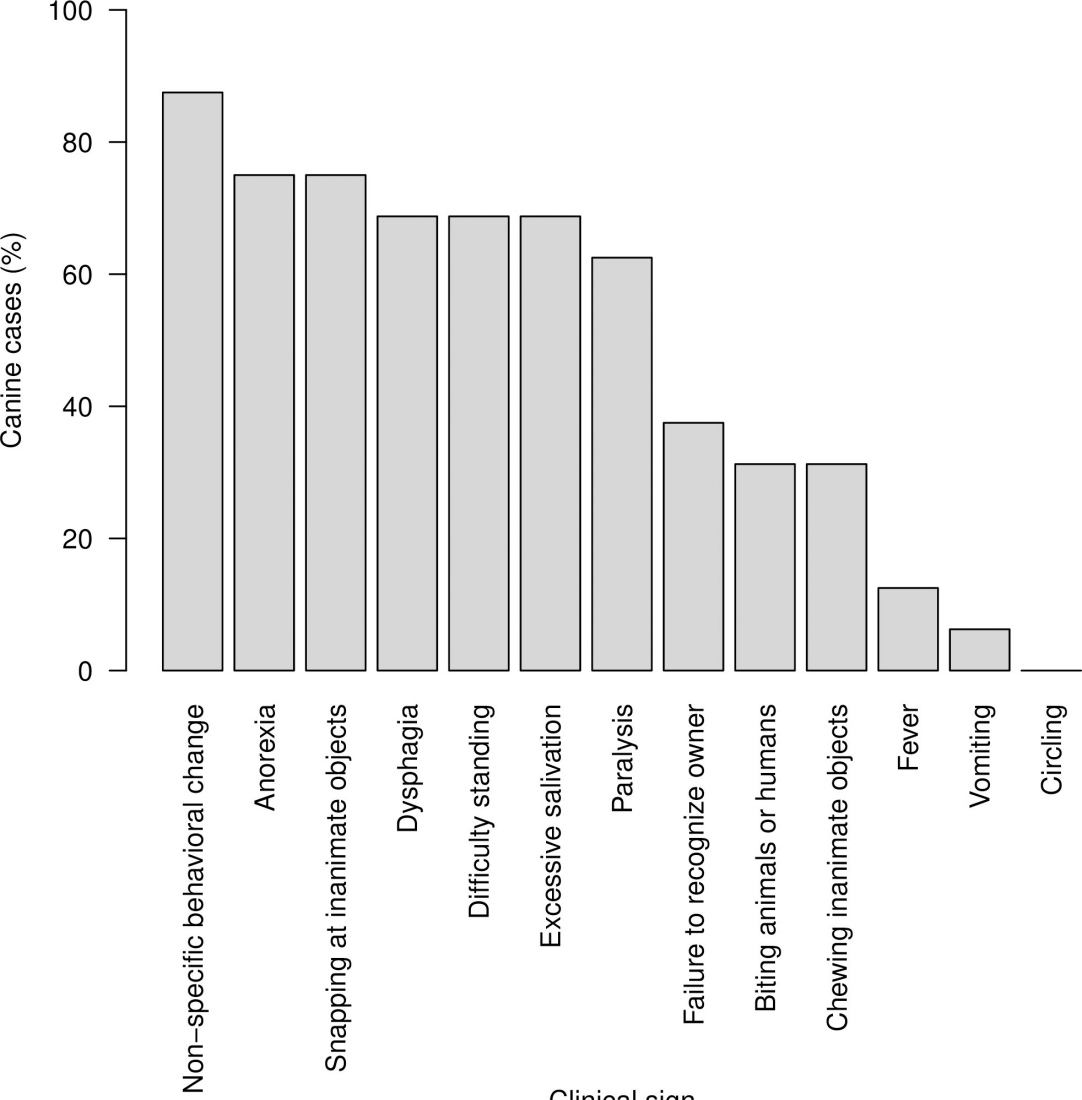
Clinical signs observed in 20 bovines with clinically suspected or laboratory confirmed rabies, reported in a study of rabies incidence in animals in selected subdistricts of Punjab, India, between August 15, 2016 and August 14, 2017.

Clinical signs associated with canine (n = 16) and bovine (n = 20) rabies cases are presented in Figs 4 and 5, respectively.

A change in behavior was the most frequently observed clinical sign in dogs (n = 14; 87.5%; Fig 4). Circling was not observed in dogs. In bovines, dysphagia, hyper-salivation and anorexia were the most frequently observed clinical signs (n = 19; 95%; Fig 5).

### Case histories

Of the 38 suspected/confirmed rabies cases, 13 were stray animals which spent most of their time in the open. Of the remaining 25, a high proportion of cases (68%; 17/25) were reported from animals that spent at least some time outside the house or farm boundary area. For rabies cases for which the history of exposure was available (32%; 12/38), most were reported to be exposed to stray dogs (92%; 11/12) followed by exposure to pet dogs (8.3%; 1/12). The route of exposure was reported to be via a bite in 83% (10/12) of the cases. For two cases (17%, 2/12), it was reported that the dogs were inside a wire-mesh cage and a rabid dog contacted them from outside.

Some of the rabies-infected domestic and pet animals in this study (16%; 4/25) had received rabies vaccination previously, but the recommended annual vaccination schedules were not subsequently followed. In 56% (14/25) of the rabies cases, the animal owner noticed a bite wound on the animal’s body but only 30.7% (4/13) of the owners requested veterinary treatment or vaccination of the animal. All of these animals died before completing the course of post-exposure rabies prophylaxis vaccination.

A total of 20 humans were exposed to rabid cases sufficiently for potential rabies virus transmission (Table 5).

**Table 5:** Exposure of humans from reported rabies cases in four sub-districts in Punjab, India, from August 15, 2016 to August 14, 2017 (PEP = post-exposure prophylaxis, HRIG = Human Rabies Immune Globulin).

Age group	Source of exposure	Number of humans exposed	Category of exposure [23]			Number of persons taking HRIG after bite of rabid animal	Number of people taking full recommended PEP
			III	II	I		
Male (< 18 years yrs)	Rabid dog	3	2	0	1	0	3
Female (< 18 years yrs)	Rabid dog	1	1	0	0	1	1
Male (> 18 yrs)	Rabid dog	7	5	1	1	1	7
Female (> 18 yrs)	Rabid cat	3	3	0	0	0	3
Male (>18yrs)	Rabid cow	3	2	0	1	0	3
Male (> 18yrs)	Rabid buffalo	2	2	0	0	0	2
Male (> 18 yrs)	Rabid mule (equine)	1	0	0	1	0	1
**Total**		**20**	**15**	**1**	**4**	**2**	**20**

Of those exposed, most (n = 13; 65%) were adult males (>18 years). Most exposed individuals received a type III exposure via a bite (n = 15; 75%), 1 (5%) received a type II exposure and the remaining 4 (20%) received a type I exposure, i.e. they were exposed through touching or licking by the animal on intact skin. All exposed humans received PEP, which included human rabies immunoglobulin (HRIG) in two cases. All exposed humans were alive at the time of completion of the study.

The confirmed rabies cases were observed to expose 11 other animals − 9 dogs, one cow and one buffalo. The route of exposure was reported to be via a bite in 10 of the 11 cases and via the mucous membrane in one of cases. Almost all exposures were to a rabid dog (10/11; 91%) and one (9%; 1/11) followed exposure to a rabid cat (Table 6).

**Table 6:** Source of exposure and treatment of 11 exposed animals from confirmed rabies cases in selected sub-districts in Punjab, India, from August 15, 2016 to August 14, 2017 (MM = Mucous membrane, PEP = post- exposure prophylaxis).

Species exposed	Source of exposure	Number exposed	Exposure via bite	(MM exposure to infectious saliva	Complete PEP	In-complete PEP	No PEP
Dog	Rabid dog (3)	8	7	1	1	0	7
Cow	Rabid dog (1)	1	1	0	1	0	0
Buffalo	Rabid dog (1)	1	1	0	0	1	0
Dog	Rabid cat (1)	1	1	0	1	0	0
Total		11	10	1	3	1	7

## Discussion

This is the first estimation of rabies incidence in canine and livestock populations in Punjab, India. We report an incidence of 2.71 and 2.03 / 10,000 dog years in pet and stray dogs, respectively. Although there are no data available about the incidence of rabies in dogs in India, a much higher annual canine rabies incidence of 41.28 per 10,000 population in Ethiopia [3] and 14 per 10,000 population in Chad [24] have been reported. These differences might be due to socio-economic, demographic and reporting factors. Alternatively, our incidence might be an underestimate of the actual rabies incidence in dogs in Punjab, India because more rigorous follow-up procedures were implemented in Ethiopia by recruitment of resident enumerators for data collection in the selected areas [3]. Interestingly, annual incidence of human rabies in Ethiopia (0.23 per 10,000; [3]) and India (0.20 per 10,000; [11]) are very similar, despite the large difference in estimated canine rabies incidence. This might suggest appropriate treatment of exposed humans [24, 25], supported by our study results.

Estimated rabies incidence did not differ significantly between stray and pet dogs in this study, in contrast to that of Kayali et al. 2003 [24] who reported 86% more rabies cases in free-roaming dogs than in confined or partially confined animals. This could be due to cultural differences: pet dogs are not always confined in rural areas of Punjab and often interact with stray dogs. Higher incidence rate of rabies in stray cattle found in this study was expected because of their free-roaming behavior in both village and urban areas. Both stray cattle and stray dogs are mostly dependent on food sources associated with the human population and therefore, are likely to have an overlapping home range. Cattle or buffaloes have previously been reported to be more likely to be bitten when kept in streets and open ground areas in Punjab [26]. However, we are not aware of any scientific investigation conducted to investigate stray cattle and dog home ranges and further investigations are required to fully elucidate this association.

This study indicated that 98 buffaloes, 56 domestic cattle, 128 pet dogs and 18 equines could be reported with rabies annually in Punjab, India − with stray cattle (96) and stray dogs (62) being important contributors − if surveillance was enhanced as it was in the current study. This is much higher than the number reported in passive disease surveillance data [16], in which 130 dogs, 95 buffalo, 78 cattle and 13 equine rabies cases were reported during a 10-year study period in Punjab, India. This indicates that rabies monitoring and surveillance programs need to be strengthened in India by involving community workers [27]. Similarly, we recommend educating, training and involving students, farmers or other suitable representatives at village- and ward-level to improve syndromic reporting of suspected rabies cases to veterinary authorities, followed by mandatory recording and reporting of rabies cases in the national veterinary database systems. The reported clinical signs of rabies in all animals in the current study are consistent with a previous study [28], except for higher proportions of anorexia, hypersalivation and ataxia (in bovines), possibly due to most animals reported being evaluated by veterinarians. We found that only 31% of the owners of the rabid animals who noted a bite on their animal consulted a veterinarian, reflecting generally limited dog care and management practices in India [13]. Improving syndromic surveillance and reporting of cases to appropriate animal and public health staff will help inform control programs and will be beneficial in control program evaluations. The burden of rabies in animal populations in India is largely unknown. Differences in geography, cultural practices, and socio-economic diversity between different states prevented us from extrapolating this data to the country level.

The current study has some limitations. Reporting of a suspected rabies case was dependent on contact between farmers and veterinary doctors and pharmacists. Therefore, under-reporting of disease incidence is likely. To overcome this potential problem, regular contact with the village-head in the selected sub-districts was maintained. We involved animal health workers and obtained laboratory confirmation of 79% of the suspected clinical rabies cases. This was possible due to laboratory submission of samples by the researchers involved in the current study.

Study results indicate that rabies incidence in animals is much higher than that previously reported from passive surveillance data in Punjab, India, particularly in the dog and stray cattle populations. The estimates in this study represent incidence of reported clinical and laboratory cases following enhanced surveillance, but likely still underestimate the true incidence of rabies in animals. We recommend community-based surveillance to enhance observation and reporting, to support the urgent need to develop and implement rabies prevention and control programs in these populations and eliminate human rabies in India.

## Supporting information

S1 Table The number of rabies-infected cases confirmed from sub-districts from 2004–2014 by the Rabies Diagnostic Laboratory, Guru Angad Dev Veterinary & Animal Sciences University, Ludhiana [16]. (DOCX)

S2 Table Livestock, owned and stray populations in the selected areas of India. (DOCX)

S1 Appendix Study questionnaire (in English). (PDF)

S2 Appendix Study questionnaire (in Punjabi). (PDF)
