# Supplementary material for: Estimation of the incidence of animal rabies in Punjab, India
Source: PLoS One. 2019 Sep 9;14(9):e0222198. doi: 10.1371/journal.pone.0222198 (PMC6733466; doi:10.1371/journal.pone.0222198)
Supplement: S1 Table — (DOCX) [file pone.0222198.s001.docx]

| **Number of confirmed cases** | **Sub-district** |
| --- | --- |
| 0 | Balachaur, Banga, Bhulath, Budhlada, Dera Baba Nanak, Kotkapura, Mukerian, Patran, Samana, Sardulgarh, Sultanpur Lodhi, Sunam, Amritsar-2, Baba Bakala, Batala, Dasua, Dharamkot, Gurdaspur, Jalalabad, Jalandhar-2, Khadur Sahib, Lehra, Mansa, Maur, Nangal, Phagwara, Talwandi Sabo |
| 1 | Gidderbaha, Patti, Guru harsahai, Fazilka, Tarn taran, Ambala, Muktsar, Garhshankar, Nakodar |
| 2 | Hoshiarpur, Abohar, Malerkotla, Malout, Dhuri, Moonak, Faridkot, Anandpur sahib, Ajnala, Nawanshahr, Tapa, Amritsar-1, Kapurthala, Sangrur |
| 3 | Dera bassi, Shahkot, Baghapurana, Khanna, Jaitu |
| 4 | Khamanon, Phull, Bassi Pathana, Phillaur |
| 5 | Kharar, Mohali, Nabha, Payal, Amloh |
| 6 | Samrala, Rajpura, Zira |
| 7 | Ropar |
| 8 | Bathinda, Patiala |
| 9 | Nihal Singh Wala, Fategharh sahib, Ferozpur, Raikot |
| 11 | Jagraon |
| 14 | Chamkaur Sahib |
| 15 | Moga |
| 16 | Barnala, Ludhiana West |
| 46 | Ludhiana East |
