# Supplementary material for: Estimation of the incidence of animal rabies in Punjab, India
Source: PLoS One. 2019 Sep 9;14(9):e0222198. doi: 10.1371/journal.pone.0222198 (PMC6733466; doi:10.1371/journal.pone.0222198)
Supplement: S2 Table — (DOCX) [file pone.0222198.s002.docx]

|  | **Livestock** | | |  | **Pet** |  | **Stray** | | **References** |
| --- | --- | --- | --- | --- | --- | --- | --- | --- | --- |
|  | Cattle | Buffalo | Equine |  | Dogs |  | Dogs | Cattle |  |
| **Sub-district(s)** |  |  |  |  |  |  |  |  |  |
| Ludhiana East | 51308 | 107838 | 774 |  | 17526 |  | 11503 | 2258 | Deputy Director Department of Animal Husbandry Ludhiana (2017) |
| Phull | 36238 | 81326 | 680 |  | 7748 |  | *6970 | *1673 | Deputy Director Department of Animal Husbandry Bathinda (2017) |
| Nihal Singh Wala | 20360 | 40080 | *576 |  | 5899 |  | *6249 | *1487 | Deputy Director Department of Animal Husbandry Moga (2017) |
| Kotkapura | 19565 | 28191 | 287 |  | 4175 |  | 1201 | 928 | Deputy Director Department of Animal Husbandry Faridkot (2017) |
| **District (s)** |  |  |  |  |  |  |  |  |  |
| Ludhiana | 203980 | 467439 | 4157 |  | 53359 |  | 31723 | 6252 | [23, 24] |
| Bathinda | 122361 | 259111 | 2022 |  | 22944 |  | 12094 | 8078 | [23, 24] |
| Moga | 108267 | 225310 | 1773 |  | 30581 |  | 8979 | 4090 | [23, 24] |
| Faridkot | 69367 | 124006 | 1688 |  | 16289 |  | 5688 | 3228 | [23, 24] |
|  |  |  |  |  |  |  |  |  |  |
| *Punjab* | *2427714* | *5159734* | *40933* |  | *470558* |  | *305482* | *100991* | [23, 24] |
|  |  |  |  |  |  |  |  |  |  |
| India | 190904110 | 108702120 | 1186450 |  | 11673000 |  | 18099800 | 5187777 | [23, 24] |

*calculation (see text)
