# Supplementary material for: Estimation of the incidence of animal rabies in Punjab, India
Source: PLoS One. 2019 Sep 9;14(9):e0222198. doi: 10.1371/journal.pone.0222198 (PMC6733466; doi:10.1371/journal.pone.0222198)
Supplement: S1 Appendix — (PDF) [file pone.0222198.s003.pdf]

School of Public Health and Zoonoses
GADVASU, Ludhiana

APPENDIX I

Identification Code …………………….. **Date…………………..**

PART I: FOR OWNER

A.PERSONNEL INFORMATION

1. Name and village/town/city of the owner ______________________________

2. Gender of the owner Male ☐ Female ☐

3. Age of the respondent: _____ years

4. Education level/qualification of the respondent`

   No formal education ☐ Primary ☐ Middle ☐ Matriculation ☐

   Secondary ☐ Undergraduate ☐ Graduate ☐

5. Members in the house (house hold size)?

	Male	Female
Number of children (less than 18 years old)		
Number of Adults (above 18 years old)		

6. Number of person involved in animal (livestock/pet) care? ☐

7. Do you keep an additional livestock attendant at your farm?

   Yes ☐ No ☐

8. Total number of animals reared by your family? ☐

   Dog ☐ Cat ☐ Cow ☐ Buffalo ☐

   Sheep ☐ Goat ☐ Horse ☐ Pig ☐

   Other ☐

B. CASE INFORMATION (SUSPECTED FOR RABIES)

1. Date when first cl. sign noticed ☐ Date of death ☐

2. Address/Location Where Found: ☐

3. Stray ☐ Wild ☐ Domestic ☐

4. Species and breed of animal ☐

5. Gender Male ☐ Female ☐

6. Age (Rough estimate for stray/wild) Months ☐ Years ☐

7. Cause of Death Euthanized ☐ Died in quarantine ☐ Killed how? ☐

8. Clinical signs

☐ Intake of inedible object	☐ Salivation	☐ Cough
☐ Diarrhoea	☐ Vomiting	☐ Fever
☐ Snapped at people/object	☐ Persons Bitten	☐ Circling
☐ Behavioural change	☐ Recognized owner	☐ Off feed
☐ Difficulty in standing/ walking	☐ Paralysis	☐ Difficult intake

Any other relevant information ☐

9. Clinical Impressions

Dumb ☐ Furious ☐ other ☐

10. Was the complete course of vaccination completed, before exposure to rabid animal?

Yes ☐ No ☐ Do not know ☐

If **yes,** did you vaccinate with booster doses of rabies every year?

Yes ☐ No ☐ Do not know ☐

11. Was there any history of exposure from any rabid animal? If Yes, answer below:

	**Yes**	**No**	**Not sure**
Through bite			
Direct contact of fresh wound with infectious saliva			
Direct contact of mucosal (eye, nose, mouth) surface with infectious saliva			
Direct contact of scratches or abrasions without bleeding with infectious saliva			
Consumption of raw milk of rabid animal **(In case of young animals)**			

If **yes, exposed** from which animal? ☐

12. How much time before your animal got exposed from suspected rabid animal? ☐

13. when you noticed animal bite/exposure to your animal from rabid animal, did you informed to veterinary hospital for post prophylaxis? Yes ☐ No ☐

If, **yes** how much doses were given? ☐

14. Did you observe wound /scratch/lameness in your animal?

Yes ☐ No ☐ Not sure ☐

15. Did you notice any bite wound on your animal?

Yes ☐ No ☐ Not sure ☐

16. Was your animal escaped from house for one or two days?

Yes ☐ No ☐

17. Did your animal was ever bitten by dog/ cat/ mongoose (wild animal)?

Yes ☐ No ☐ Not sure ☐

If **Yes**, did you reported to veterinary hospital. Yes ☐ No ☐

18. If you don't know about questions **11-17**, what do you think was the reason of rabies at your farm?

☐

C. HUSBANDRY PRACTICES

19. Was a Rabies case reported on your farm since last 5 years? Yes ☐ No ☐

If yes, in which species of the animal it was? ☐

20. How did you keep your livestock/pet?

a) Free to roam around the house compound, village/ town? Yes ☐ No ☐

If **Yes** for how much time (hours)? 0-6 ☐ 12-18 ☐ 12-18 ☐ 18-24 ☐

b) Tie outside in open area (e.g. street, open land etc.)? Yes ☐ No ☐

If **Yes** for how much time (hours)? 0-6 ☐ 12-18 ☐ 12-18 ☐ 18-24 ☐

c) Free to roam inside a well-marked boundary wall area? Yes ☐ No ☐

If **Yes** for how much time (hours)? 0-6 ☐ 12-18 ☐ 12-18 ☐ 18-24 ☐

d) Tie inside in well boundary wall area? Yes ☐ No ☐

If **Yes** for how much time (hours)? 0-6 ☐ 12-18 ☐ 12-18 ☐ 18-24 ☐

e) Tie inside a partial boundary wall/fencing? Yes ☐ No ☐

If **Yes** for how much time (hours)? 0-6 ☐ 12-18 ☐ 12-18 ☐ 18-24 ☐

21. Was your livestock/pet easily accessible to stray dogs? Yes ☐ No ☐

22. Have you ever seen your animal in contact with stray dogs? Yes ☐ No ☐

23. Have you ever seen Mongoose/Wild animal in your village/ward? Yes ☐ No ☐

If **Yes**, do you believe they can bite your animal? Yes ☐ No ☐

D. RISK OF ANIMAL EXPOSURE FROM SUSPECTED RABID ANIMAL

24. Did any other animal come in contact with this rabid animal? Yes ☐ No ☐
(If No, do not go for next Questions)

25. How many animals come in contact with rabid animal? ☐

26. What type of animals come in contact with rabid animal?

☐ Dog ☐ Cat ☐ Cow

☐ Buffalo ☐ Sheep ☐ Goat

☐ Pig Other ☐

Detailed information related to exposed animals?

**Detail Performa for exposed animal (M=Male, F=Female)**											
**Sr. No.**	**Species of animal**	**Breed**	**Young**		**Adult**					**Chose type of contact***	**No. of Post-Prophylaxis doses used**
			**M**	**F**	**M**	**Female**					
						**Pregnant**		**lactating**			
						**Yes**	**No**	**Yes**	**No**		
1.											
2.											
3.											
4.											
5.											
6.											
7.											
8.											
9.											
10.											

*** Mention type of contact**	**Number**
Touching or licks on intact skin	1
Nibbling of uncovered skin, minor scratches or abrasions without bleeding	2
Direct contact of abrasions without bleeding or scratches with infectious saliva	3

Direct contact of mucosal (eye, nose, mouth) surface with infectious saliva	4
Direct contact of fresh wound with infectious saliva	5
Single or multiple transdermal bites	6
Consumption of raw milk of rabid animal	7

E. RISK OF HUMAN EXPOUSORE

27. Did you, your family member or other person come in contact with rabid animal? Yes ☐ No ☐

(If No, do not go for next Questions)

28. How many people came in contact with rabid animal? ☐

29. Detail information related to exposed humans? **(Please provide detailed information)**

**Detail Performa for exposed human (M=Male, F=Female)**																
**Sr. No.**	**Young**		**Adult**										**Chose type of contact***	**No. of Post-Prophylaxis vaccination doses consumed**	**Rabies immunoglobulin consumed**	
	**M**	**F**	**Male**		**Female**											
			**Working**		**Pregnant**		**Breast feeding**		**Working**						**Yes**	**No**
			**Yes**	**No**	**Yes**	**No**	**Yes**	**No**	**Yes**	**No**						
1.																
2.																
3.																
4.																
5.																
6.																
7.																
8.																
9.																
10																

*** Mention type of contact**	**Number**
Touching or licks on intact skin	1
Nibbling of uncovered skin, minor scratches or abrasions without bleeding	2
Direct contact of abrasions without bleedingor scratches with infectious saliva	3
Direct contact of mucosal (eye, nose, mouth) surface with infectious saliva	4
Direct contact of fresh wound with infectious saliva	5
Single or multiple transdermal bites when the animal was provoked	6
Single or multiple transdermal bites without provoking the animal	7
Consumption of raw milk of rabid animal	8

30. Name and address of exposed persons?

PART II: FOR VETS / PARAVETS

PERSONAL INFORMATION

Owner Information ID (if any): ☐

1. Name ☐
2. Name of CVH / CVD ☐
3. Pin code ☐
4. Designation? Veterinary doctor ☐ Pharmacist (VI) ☐
5. Age _____ years
6. Gender Male ☐ Female ☐
7. Years in veterinary practice? ☐
8. How many cases of rabies you seen in your veterinary practice? ☐
9. Did you ever get exposure of rabies? Yes ☐ No ☐

If, yes **Chose type of contact***

* **Mention type of contact**	**Number**
Touching or licks on intact skin	1
Nibbling of uncovered skin, minor scratches or abrasions without bleeding	2
Direct contact of abrasions without bleeding or scratches with infectious saliva	3
Direct contact of mucosal (eye, nose, mouth) surface with infectious saliva	4
Direct contact of fresh wound with infectious saliva	5
Single or multiple transdermal bites when the animal was provoked	6
Single or multiple transdermal bites without provoking the animal	7
Consumption of raw milk of rabid animal	8

B. QUESTIONS RELATED SUSPECTED RABID ANIMAL

10. Species of animal ☐
11. Age _____
12. Sex Male ☐ Female ☐

13. Cause of Death Euthanized ☐ Died in quarantine ☐ Killed how? ☐

14. Clinical signs

☐ Intake of inedible object	☐ Salivation	☐ Cough
☐ Diarrhoea	☐ Vomiting	☐ Fever
☐ Snapped at people/object	☐ Persons Bitten	☐ Circling
☐ Behavioural change	☐ Recognized owner	☐ Off feed
☐ Difficulty in standing/ walking	☐ Paralysis	☐ Difficult intake

Any other relevant information ☐

15. Clinical Impressions

Dumb ☐ Furious ☐ Don’t know ☐

16. Based on clinical symptoms, do you think it is a case of rabies?

Yes ☐ No ☐ Not sure ☐

If, **not sure** do you think it could be due to any other disease? Yes ☐ No ☐

If, Yes which disease it could be?

☐

C. RELATED TO OCCUPATIONAL EXPOSURE

17. Are you vaccinated for rabies? Yes ☐ No ☐

18. Do you take booster doses of rabies vaccine every year? Yes ☐ No ☐

19. Have you ever checked your antibody titre for rabies antibodies? Yes ☐ No ☐

20. Do you take personal protective equipment while handling a suspected rabies case? Yes ☐ No ☐

If, **yes** chose one or more personal protective equipment

Gloves ☐ Surgical mask ☐ Protective goggles ☐

Gowns or Lab. coats ☐ Other equipment ☐

None of these ☐

21. Do you come in contact with the current rabid animal? Yes ☐ No ☐

If, yes **Chose type of contact***

* **Mention type of contact**	**Number**
Touching or licks on intact skin	1
Nibbling of uncovered skin, minor scratches or abrasions without bleeding	2
Direct contact of abrasions without bleeding or scratches with infectious saliva	3
Direct contact of mucosal (eye, nose, mouth) surface with infectious saliva	4
Direct contact of fresh wound with infectious saliva	5
Single or multiple transdermal bites when the animal was provoked	6
Single or multiple transdermal bites without provoking the animal	7
Consumption of raw milk of rabid animal	8
