# Supplementary material for: Estimation of the incidence of animal rabies in Punjab, India
Source: PLoS One. 2019 Sep 9;14(9):e0222198. doi: 10.1371/journal.pone.0222198 (PMC6733466; doi:10.1371/journal.pone.0222198)
Supplement: S2 Appendix — (PDF) [file pone.0222198.s004.pdf]

ਸਕੂਲ ਆਫ ਪਬਲਿਕ ਹੈਲਥ ਐਂਡ ਜ਼ੂਨੌਸਿਸ
ਗੱਡਵਾਸੂ, ਲੁਧਿਆਣਾ

ਅੰਤਿਕਾ I

ਪਛਾਣ ਕੋਡ .... **ਮਿਤੀ.......................**

ਭਾਗ I: ਮਾਲਕ ਦੇ ਲਈ

ੳ. ਵਿਅਕਤੀਗਤ ਜਾਣਕਾਰੀ

1. ਜਵਾਬਦੇਹ ਦਾ ਨਾਮ ਅਤੇ ਪਿੰਡ/ ਕਸਬੇ / ਸ਼ਹਿਰ ਦਾ ਪਤਾ ____________________

2. ਝਵਾਬਦੇਹ ਦਾ ਲਿੰਗ   ਪੁਰਸ਼ ☐   ਇਸਤਰੀ ☐

3. ਝਵਾਬਦੇਹ ਦੀ ਉਮਰ: ______ ਸਾਲ

4. ਜਵਾਬਦੇਹ ਦੀ ਸਿੱਖਿਆ ਦਾ ਪੱਧਰ/ਯੋਗਤਾ

   ਕੋਈ ਵਿਦਿਅਕ ਯੋਗਤਾ ਨਹੀਂ ☐   ਪੰਜਵੀਂ ☐   ਅਠਵੀਂ ☐   ਦਸਵੀਂ ☐

   ਬਾਰਵੀਂ ☐   ਗ੍ਰੈਜੂਏਟ ਦੇ ਤਹਿਤ ☐   ਗ੍ਰੈਜੂਏਟ ☐

5. ਘਰ ਵਿੱਚ ਰਿਹਣ ਵਾਲੇ ਮੈਂਬਰਾ ਦੀ ਗਿਣਤੀ ?

	ਪੁਰਸ਼	ਇਸਤਰੀ
ਕੁੱਲ ਬਚਿਆ ਦੀ ਗਿਣਤੀ (ਉਮਰ 18 ਸਾਲ ਤੋਂ ਘੱਟ)		
ਕੁੱਲ ਵਡਿਆ ਦੀ ਗਿਣਤੀ (ਉਮਰ 18 ਸਾਲ ਤੋਂ ਵੱਧ)		

6. (ਪਸ਼ੂ ਧਨ/ ਪਾਲਤੂ ਜਾਨਵਰਾਂ) ਦੀ ਸਾਂਭ ਸੰਭਾਲ ਕਰਨ ਵਾਲੇ ਕੁੱਲ ਵਿਅਕਤੀਆਂ ਦੀ ਗਿਣਤੀ ? ☐

7. ਕੀ ਤੁਸੀਂ ਪਸ਼ੂਆਂ ਦੀ ਸਾਂਭ ਸੰਭਾਲ ਲਈ ਕੋਈ ਸੇਵਕ ਰਖਿਆ ਹੋਇਆ ਹੈ?

   ਹਾਂ ☐   No ☐

8. ਘਰ ਵਿੱਚ ਰੱਖੇ ਕੁੱਲ ਪਸ਼ੂਆਂ ਦੀ ਗਿਣਤੀ ? ☐

   ਖੁੱਤਾ ☐   ਬਿੱਲੀ ☐   ਗਾਂ ☐   ਮੱਝ ☐

   ਭੇਡ ☐   ਬੱਕਰੀ ☐   ਘੋੜਾ ☐   ਸੂਰ ☐

   ਹੋਰ ____________________

ਅ. ਬਿਮਾਰ ਪਸ਼ੂ ਬਾਰੇ ਜਾਣਕਾਰੀ (ਹਲਕਾ ਦੇ ਸ਼ੱਕ ਸੰਬਧੀ)

1. ਤਰੀਖ ਜਿਸ ਦਿਨ ਬਿਮਾਰੀ ਦਾ ਪਹਿਲਾ ਲਛਣ ਦੇਖਿਆ ☐ ਮੌਤ ਦੀ ਤਰੀਖ ☐

2. ਪੱਤਾ/ਜੱਗ੍ਹਾ ਤੇ ਮਿਲਿਆ: ☐

3. ਅਵਾਰਾ ☐ ਜੰਗਲੀ ☐ ਘਰੇਲੂ ☐

4. ਜਾਨਵਰ ਦੀ ਜਾਤੀ ਅਤੇ ਨਸਲ ☐

5. ਲਿੰਗ ਪੁਰਸ਼ ☐ ਇਸਤਰੀ ☐

6. ਉਮਰ (ਅਵਾਰਾ / ਜੰਗਲੀ ਜਾਨਵਰਾਂ ਲਈ ਅੰਦਾਜ਼ਾ ਲਗਾਓ) ਮਹੀਨੇ ☐ ਸਾਲ ☐

7. ਮੌਤ ਦਾ ਕਾਰਣ ਯੁਥੇਨਾਈਜ਼ ☐ ਹਲਕਾ ਦੋਰਾਨ ਮੌਤ ☐ ਮਾਰਨਾ (ਕਿੱਸ ਤਰ੍ਹਾਂ ਮਾਰਿਆ)? ☐

8. ਬਿਮਾਰੀ ਦੇ ਲਛਣ

☐ ਨਾ ਖਾਣ ਯੋਗ ਵਸਤਾਂ ਨੂੰ ਖਾਣਾ	☐ ਮੂੰਹ ਵਿੱਚੋਂ ਝੱਗ ਆਉਣਾ	☐ ਖੰਘ
☐ ਧੱਸਤ ਆਉਣੇ	☐ ਉਲਟੀ ਆਉਣਾ	☐ ਬੁਖਾਰ
☐ ਲੋਕਾ/ਚੀਜ਼ਾਂ ਤੇ ਦੰਦੀ ਵੱਢਣ ਦੀ ਕੋਸ਼ਿਸ਼ ਕਰਨੀ	☐ ਵਿਅਕਤੀਆਂ ਨੂੰ ਵੱਢਿਆ ਗਿਆ	☐ ਘੁੱਮਣਾ
☐ ਸੁਬਾਹ ਵਿੱਚ ਤੱਬਦੀਲੀ	☐ ਮਾਲਕ ਨੂੰ ਪਛਾਣਨਾ	☐ ਭੁੱਖਾ
☐ ਖੱੜ ਹੋਣ/ਚਲਣ ਵਿੱਚ ਤਕਲੀਫ	☐ ਅਧਰੰਗ	☐ ਖਾਣ ਵਿੱਚ ਤੱਕਲੀਫ

ਕੋਈ ਵੀ ਹੋਰ ਸੰਬੰਧਿਤ ਜਾਣਕਾਰੀ ☐

9. ਕਲੀਨਿਕਲ ਕਿਸਮ

ਗੂੰਗਾ ☐ ਭੱੜਕਾਓ ☐ ਹੋਰ ☐

10. ਹਲਕੇ ਹੋਏ ਜਾਨਵਰ ਦੇ ਸਿੱਧੇ ਸੰਮਪਰਕ ਵਿੱਚ ਆਉਣ ਤੋਂ ਪਹਿਲਾ ਕੀ ਟੀਕਾਕਰਣ ਦਾ ਪੂਰਾ ਕੋਰਸ ਪੂਰਾ ਹੋ ਚੁਕਿਆ ਸੀ ?

ਹਾਂ ☐ ਨਹੀਂ ☐ ਪੱਤਾ ਨਹੀਂ ☐

ਜੇ ਹਾਂ, ਤਾਂ ਕੀ ਤੁਸੀਂ ਹਰ ਸਾਲ ਹਲਕਾ ਦੇ ਟੀਕੇ ਦੀ ਬੂਸਟਰ ਡੋਜ਼ ਲਗਾਉਂਦੇ ਸੀ ?

ਹਾਂ ☐ ਨਹੀਂ ☐ ਪੱਤਾ ਨਹੀਂ ☐

11. ਕੀ ਪਛੋਕੜ ਵਿੱਚ ਇਹ ਕਿਸੇ ਹਲਕੇ ਹੋਏ ਜਾਨਵਰ ਦੇ ਸਿੱਧੇ ਸੰਪਰਕ ਵਿੱਚ ਅਇਆ ਸੀ ? ਜੇ ਹਾਂ ਤਾਂ,

ਥੱਲੇ ਜਵਾਬ ਦਿਓ:

	**ਹਾਂ**	**ਨਹੀਂ**	**ਪੱਤਾ ਨਹੀਂ**
ਵੱਢਣ ਨਾਲ			
ਤਾਜੇ ਜਖਮ ਦਾ ਹਲਕੇ ਹੋਏ ਜਾਨਵਰ ਦੇ ਥੁੱਕ ਦੇ ਸਿੱਧੇ ਸੰਪਰਕ ਵਿੱਚ ਆਓਣ ਨਾਲ			
ਅੱਖ, ਨੱਕ ਜਾਂ ਮੂੰਹ ਦਾ ਹਲਕੇ ਹੋਏ ਜਾਨਵਰ ਦੇ ਥੁੱਕ ਦੇ ਸਿੱਧੇ ਸੰਪਰਕ ਵਿੱਚ ਆਓਣ ਨਾਲ			
ਝਰੀਟ, ਜਾਂ ਬਿਨਾਂ ਖੁਨ ਵਗੇ ਜਖਮ ਦਾ ਹਲਕੇ ਹੋਏ ਜਾਨਵਰ ਦੇ ਥੁੱਕ ਦੇ ਸਿੱਧੇ ਸੰਪਰਕ ਵਿੱਚ ਆਓਣ ਨਾਲ			
ਹਲਕੇ ਹੋਏ ਜਾਨਵਰ ਦੇ ਕੱਚੇ ਦੁੱਧ ਦਾ ਸੇਵਣ ਕਰਨ ਨਾਲ			

ਜੇ ਹਾਂ ਤਾਂ, ਕਿਸ ਜਾਨਵਰ ਦੇ ਸੰਪਰਕ ਆਇਆ ਸੀ ?

12. ਤੁੱਹਾਡਾ ਜਾਨਵਰ ਕਿਨਾ ਸਮਾਂ ਪਹਿਲਾ ਸ਼ੱਕੀ ਹਲਕੇ ਜਾਨਵਰ ਦੇ ਸਿੱਧੇ ਸੰਪਰਕ ਵਿੱਚ ਆਇਆ ਸੀ?

13. ਜਾਨਵਰ ਦੇ ਦੰਦ ਵੱਜਣ/ ਹਲਕੇ ਹੋਏ ਜਾਨਵਰ ਦੇ ਸੰਪਰਕ ਵਿੱਚ ਅਉਣ ਤੇ , ਕੀ ਤੁਸੀਂ ਹਲਕਾ ਸੰਬਧੀ ਟੀਕਾਕਰਨ ਕਰਨ ਲਈ ਪਸ਼ੂ ਹਸਪਤਾਲ ਵਿੱਚ ਸੰਪਰਕ ਕਿੱਤਾ ਸੀ? ਹਾਂ ☐ ਨਹੀਂ ☐

ਜੇ ਹਾਂ ਤਾਂ, ਕਿਨ੍ਹੇ ਟੀਕੇ ਲਗਵਾਏ ਗਏ?

14. ਕੀ ਤੁਸੀਂ ਅਪਣੇ ਪਸ਼ੂ ਵਿੱਚ ਕੋਈ ਜਖਮ/ ਝਰੀਟ/ਲੰਗੜਾਪਨ ਦੇਖਿਆ ਸੀ?

ਹਾਂ ☐ ਨਹੀਂ ☐ ਪੱਤਾ ਨਹੀਂ ☐

15. ਕੀ ਤੁਸੀਂ ਅਪਣੇ ਪਸ਼ੂ ਵਿੱਚ ਕੋਈ ਦੰਦੀ ਵੱਢਣ ਨਾਲ ਹੋਏ ਜਖਮ ਨੂੰ ਦੇਖਿਆ ਸੀ?

ਹਾਂ ☐ ਨਹੀਂ ☐ ਪੱਤਾ ਨਹੀਂ ☐

16. ਕੀ ਤੁਹਾਡਾ ਪਸ਼ੂ ਇੱਕ ਜਾ ਦੋ ਦਿਨਾਂ ਲਈ ਘਰ ਤੋਂ ਬਾਹਰ ਰਿਹਾ ਸੀ ?

ਹਾਂ ☐ ਨਹੀਂ ☐

17. ਕੀ ਤੁਹਾਡੇ ਪਸ਼ੂ ਨੂੰ ਕੱਦੇ ਕੁੱਤੇ/ ਬਿੱਲੀ/ ਨਿਉਲਾ (ਜੰਗਲੀ ਜਾਨਵਰ) ਨੇ ਵੱਢਿਆ ਸੀ ?

ਹਾਂ ☐ ਨਹੀਂ ☐ ਪੱਤਾ ਨਹੀਂ ☐

ਜੇ ਹਾਂ ਤਾਂ, ਕੀ ਤੁਸੀਂ ਇਸ ਸੰਬਧ ਵਿੱਚ ਪਸ਼ੂ ਹਸਪਤਾਲ ਵਿੱਚ ਸੰਪਰਕ ਕਿੱਤਾ ਸੀ ? ਹਾਂ ☐ ਨਹੀਂ ☐

18. ਜੇ ਤੁਸੀਂ 11-17 ਤੱਕ ਦੇ ਪ੍ਰਸ਼ਨਾ ਦੇ ਉਤਰ ਨਹੀਂ ਦੇ ਸਕਦੇ, ਤਾਂ ਤੁਹਾਡੇ ਫਾਰਮ ਤੇ ਅਈ ਹਲਕਾ ਦੀ ਬਿਮਾਰੀ ਦਾ ਕੀ ਕਾਰਨ ਹੋ ਸਕਦਾ?

ੲ. ਪਸ਼ੂ ਪਾਲਣ ਦੀ ਵਿਧੀ ਸੰਬਧੀ

19. ਕੀ ਪਿਛਲੇ ਪੰਜ ਸਾਲਾ ਦੌਰਾਨ ਤੁਹਾਡੇ ਫਾਰਮ ਤੇ ਕੋਈ ਹਲਕਾ ਦੀ ਬਿਮਾਰੀ ਆਈ ਸੀ? ਹਾਂ ☐ ਨਹੀਂ ☐

ਜੇ ਹਾਂ ਤਾਂ, ਕਿਸ ਜਾਤੀ ਦੇ ਪਸ਼ੂ ਵਿੱਚ ਆਈ ਸੀ? ☐

20. ਤੁਸੀਂ ਅਪਣੇ ਪਸ਼ੂਆਂ/ ਪਾਲਤੁ ਜਾਨਵਰ ਨੂੰ ਕਿਸ ਤਰ੍ਹਾਂ ਰਖਦੇ ਹੋ?

ੳ) ਘਰ ਦੇ ਆਂਗਣ, ਪਿੰਡ /ਕਸਬੇ ਵਿੱਚ ਖੁੱਲਾ ਛੱਡਦੇ ਹੋ ? ਹਾਂ ☐ ਨਹੀਂ ☐

ਜੇ ਹਾਂ ਤਾਂ ਕਿੰਨੇਂ ਸਮੇਂ ਲਈ (ਘੰਟਿਆਂ ਵਿੱਚ) 0-6 ☐ 12-18 ☐ 12-18 ☐ 18-24 ☐

ਅ) ਬਾਹਰ ਖੁਲੀ ਜਗਾਹ ਵਿੱਚ ਬੰਨ੍ਹਦੇ ਹੋ)? (ਗੱਲੀ, ਖੁੱਲੀ ਜਗ੍ਹਾ ਅਦਿ) ਹਾਂ ☐ ਨਹੀਂ ☐

ਜੇ ਹਾਂ ਤਾਂ ਕਿੰਨੇਂ ਸਮੇਂ ਲਈ (ਘੰਟਿਆਂ ਵਿੱਚ) 0-6 ☐ 12-18 ☐ 12-18 ☐ 18-24 ☐

ੲ) ਸੀਮਤ ਚਾਰ ਦਿਵਾਰੀ ਵਿੱਚ ਖੁੱਲਾ ਛੱਡਦੇ ਹੋ? ਹਾਂ ☐ ਨਹੀਂ ☐

ਜੇ ਹਾਂ ਤਾਂ ਕਿੰਨੇਂ ਸਮੇਂ ਲਈ (ਘੰਟਿਆਂ ਵਿੱਚ) 0-6 ☐ 12-18 ☐ 12-18 ☐ 18-24 ☐

ਸ) ਕੰਧ ਦੀ ਸੀਮਤ ਚਾਰ ਦਿਵਾਰੀ ਵਿੱਚ ਬੰਨ੍ਹ ਕੇ ਰੱਖਦੇ ਹੋ? ਹਾਂ ☐ ਨਹੀਂ ☐

ਜੇ ਹਾਂ ਤਾਂ ਕਿੰਨੇਂ ਸਮੇਂ ਲਈ (ਘੰਟਿਆਂ ਵਿੱਚ) 0-6 ☐ 12-18 ☐ 12-18 ☐ 18-24 ☐

ਹ) ਅਧੂਰੀ ਕੰਦ/ਕੰਡਿਆਲੀ ਤਾਰ ਦੀ ਚਾਰ ਦਿਵਾਰੀ ਵਿੱਚ ਬੰਨ੍ਹ ਕੇ ਰੱਖਦੇ ਹੋ? ਹਾਂ ☐ ਨਹੀਂ ☐

ਜੇ ਹਾਂ ਤਾਂ ਕਿੰਨੇਂ ਸਮੇਂ ਲਈ (ਘੰਟਿਆਂ ਵਿੱਚ) 0-6 ☐ 12-18 ☐ 12-18 ☐ 18-24 ☐

21. ਕੀ ਤੁਹਾਡਾ ਪਸ਼ੂਧਨ ਅਸਾਨੀ ਨਾਲ ਅਵਾਰਾ ਕੁੱਤਿਆਂ ਦੀ ਪਹੁੰਚ ਵਿੱਚ ਹੈ? ਹਾਂ ☐ ਨਹੀਂ ☐

22. ਕੀ ਤੁਸੀਂ ਕਦੇ ਅਪਣੇ ਪਸ਼ੂਆਂ ਨੂੰ ਅਵਾਰਾ ਕੁੱਤਿਆਂ ਦੇ ਸੰਪਰਕ ਵਿੱਚ ਦੇਖਿਆ ਹੈ? ਹਾਂ ☐ ਨਹੀਂ ☐

23. ਕੀ ਤੁਸੀਂ ਕਦੇ ਨਿਉਲਾ/ਜੰਗਲੀ ਜਾਨਵਰ ਅਪਣੇ ਪਿੰਡ ਵਿੱਚ ਦੇਖੇ ਹਨ? ਹਾਂ ☐ ਨਹੀਂ ☐

ਜੇ ਹਾਂ ਤਾਂ ਕੀ ਤੁਸੀਂ ਮੰਨਦੇ ਹੋ ਕਿ ਉਹ ਤੁਹਾਡੇ ਪਸ਼ੂਆਂ ਨੂੰ ਵੱਢ ਸਕਦੇ ਹਨ? ਹਾਂ ☐ ਨਹੀਂ ☐

ਸ. ਸ਼ੰਕਾ ਅਧੀਨ ਹਕਕੇ ਪਸ਼ੂ ਤੋਂ ਸਿਹਤਮੰਦ ਪਸ਼ੂ ਬਿਮਾਰੀ ਲਗਣ ਦੇ ਖਤਰੇ ਸੰਬਧੀ

24. ਕੀ ਕੋਈ ਹੋਰ ਪਸ਼ੂ ਇਸ ਹਲਕੇ ਹੋਏ ਜਾਨਵਰ ਦੇ ਸਿੱਧੇ ਸੰਪਰਕ ਵਿੱਚ ਆਇਆ ਸੀ? ਹਾਂ ☐ ਨਹੀਂ ☐

(ਜੇ ਨਹੀਂ, ਤਾਂ ਅਗਲੇ ਪ੍ਰਸ਼ਨਾਂ ਦੇ ਜਵਾਬ ਨਾਂ ਦੇਓ ?)

25. ਕਿੰਨ੍ਹੇ ਜਾਨਵਰ ਹਲਕੇ ਹੋਏ ਪਸ਼ੂ ਦੇ ਸਿੱਧੇ ਸੰਪਰਕ ਵਿੱਚ ਅਏ ਹਨ? ☐

26. ਕਿਸ ਤਰ੍ਹਾਂ ਦੇ ਜਾਨਵਰ ਹਲਕੇ ਹੋਏ ਪਸ਼ੂ ਦੇ ਸਿੱਧੇ ਸੰਪਰਕ ਵਿੱਚ ਅਏ ਹਨ?

☐ ਕੁੱਤਾ ☐ ਬਿੱਲੀ ☐ ਗਾਂ

☐ ਮੱਝ ☐ ਭੇਡ ☐ ਬੱਕਰੀ

☐ ਸੂਰ   ਕੁੱਝ ਹੋਰ ☐

ਹਲਕੇ ਹੋਏ ਪਸ਼ੂ ਦੇ ਸਿੱਧੇ ਸੰਪਰਕ ਵਿੱਚ ਆਏ ਪਸ਼ੂਆਂ ਬਾਰੇ ਵਿਸਥਾਰ ਵਿੱਚ ਜਾਣਕਾਰੀ

ਹਲਕੇ ਹੋਏ ਪਸ਼ੂ ਦੇ ਸਿੱਧੇ ਸੰਪਰਕ ਵਿੱਚ ਆਏ ਪਸ਼ੂਆਂ ਦੀ ਜਾਣਕਾਰੀ ਸੰਬਧੀ ਪ੍ਰਫੋਰਮਾ (ਨ=ਨਰ, ਮ=ਮਾਦਾ)											
**ਕ੍ਰਮ ਸੰਖਿਆ**	**ਪਸ਼ੂਆਂ ਦੀ ਜਾਤੀ**	**ਨਸਲ**	**ਛੋਟੇ**		**ਵੱਡੇ**					**ਸੰਪਰਕ ਦੀ ਕਿਸਮ ਨੂੰ ਚੁਣੋ***	**ਪੋਸਟ ਪ੍ਰੋਫਾਈਲੈਕਸਿਸ ਦੀਆਂ ਡੋਜ਼ਾਂ ਦੀ ਗਿਣਤੀ**
			ਨ	ਮ	ਨ	**ਮਾਦਾ**					
						**ਗਰਭਵਤੀ**		**ਦੁੱਧ ਚੁੰਘਾਉਣ ਵਾਲੇ**			
						ਹਾਂ	ਨਹੀਂ	ਹਾਂ	ਨਹੀਂ		
1.											
2.											
3.											
4.											
5.											
6.											
7.											
8.											
9.											
10.											

*** ਸੰਪਰਕ ਦੀ ਕਿਸਮ ਬਾਰੇ ਜਿਕਰ ਕਰੋ**	**ਸੰਖਿਆ**
ਛੂਹਇਆ ਜਾਂ ਚਮੜੀ ਨੂੰ ਚਟਿੱਆ	1
ਨੰਗੀ ਚਮੜੀ ਉਤੇ ਦਵਾਬ, ਛੋਟੀ ਝਰੀਟ ਜਾਂ ਬਿਨਾਂ ਖੂਨ ਵੱਗੇ ਵਾਲਾ ਜਖਮ ਹੋਇਆ	2
ਬਿਨਾਂ ਖ਼ੂਨ ਵਗੇ ਜਖਮ ਜਾਂ ਝਰੀਟ ਦਾ ਹਲਕੇ ਹੋਏ ਜਾਨਵਰ ਦੇ ਥੁੱਕ ਦੇ ਸਿੱਧੇ ਸੰਪਰਕ ਵਿੱਚ ਆਓਣ ਨਾਲ	3
ਅੱਖ, ਨੱਕ ਜਾਂ ਮੂੰਹ ਦਾ ਹਲਕੇ ਹੋਏ ਜਾਨਵਰ ਦੇ ਥੁੱਕ ਦੇ ਸਿੱਧੇ ਸੰਪਰਕ ਵਿੱਚ ਆਓਣ ਨਾਲ	4
ਤਾਜੇ ਜਖਮ ਦਾ ਹਲਕੇ ਹੋਏ ਜਾਨਵਰ ਦੇ ਥੁੱਕ ਦੇ ਸਿੱਧੇ ਸੰਪਰਕ ਵਿੱਚ ਆਓਣ ਨਾਲ	5
ਇੱਕ ਜਾਂ ਇੱਕ ਤੋਂ ਵੱਧ ਥਾਂਵਾ ਤੇ ਵੱਡਿਆ ਹੋਵੇ ਤੇ ਜਖਮ ਹੋ ਗਏ ਹੋਣ	6
ਹਲਕੇ ਹੋਏ ਜਾਨਵਰ ਦੇ ਕੱਚੇ ਦੁੱਧ ਦਾ ਸੇਵਣ ਕਰਨ ਨਾਲ	7

ਹ. ਮਨੁੱਖਾਂ ਵਿੱਚ ਐਕਸਪੋਜਰ ਬਾਰੇ

27. ਕੀ ਤੁਸੀਂ ਜਾਂ ਤੁਹਾਡੇ ਪਰੀਵਾਰ ਦਾ ਕੋਈ ਜੀ ਹਲਕੇ ਜਾਨਵਰ ਦੇ ਸੰਪਰਕ ਵਿੱਚ ਆਇਆ ਸੀ ?   ਹਾਂ ☐   ਨਹੀਂ ☐

    (ਜੇ ਨਹੀਂ, ਤਾਂ ਅਗਲੇ ਪ੍ਰਸ਼ਨਾਂ ਦੇ ਜਵਾਬ ਨਾਂ ਦੇਓ ?)

28. ਪਰਿਵਾਰ ਦੇ ਕਿੰਨੇ ਜੀ ਹਲਕੇ ਹੋਏ ਜਾਨਵਰ ਦੇ ਸੰਪਰਕ ਵਿੱਚ ਆਏ ਸਨ? ☐

29. ਐਕਸਪੋਜਡ ਮਨੁੱਖਾਂ ਬਾਰੇ ਵਿਸਥਾਰ ਨਾਲ ਜਾਣਕਾਰੀ ? (ਕ੍ਰਿਪਾ ਕਰਕੇ ਵਿਸਥਾਰ ਨਾਲ ਜਾਣਕਾਰੀ ਦਿਓ)

ਐਕਸਪੋਜਡ ਮਨੁੱਖਾਂ ਬਾਰੇ ਵਿਸਥਾਰ ਨਾਲ ਜਾਣਕਾਰੀ ਲਈ ਪ੍ਰਫੋਰਮਾ (ਪ=ਪੁਰਸ਼, ਇ=ਇਸਤਰੀ)

ਕ੍ਰਮ ਸੰਖਿਆ.	ਬੱਚੇ		Adult								ਸੰਪਰਕ ਦੀ ਕਿਸਮ ਨੂੰ ਚੁਣੋ*	ਪੋਸਟ ਪ੍ਰੋਫਾਈਲੈਕਸਿਸ ਦੀਆਂ ਡੋਜਾਂ ਦੀ ਗਿਣਤੀ	ਇਮੁਨੋਗਲੋਬੁਲੀਨ ਲਗਾਏ	
	ਪ	ਇ	ਪੁਰਸ਼		ਇਸਤਰੀ									
			ਕੰਮ ਕਰਨ ਵਾਲੇ		ਗਰਭਵਤੀ		ਦੁੱਧ ਚੁੰਘਾਉਣ ਵਾਲੇ		ਕੰਮ ਕਰਨ ਵਾਲੇ				ਹਾਂ	ਨਹੀਂ
			ਹਾਂ	ਨਹੀਂ	ਹਾਂ	ਨਹੀਂ	ਹਾਂ	ਨਹੀਂ	ਹਾਂ	ਨਹੀਂ				
1.														
2.														
3.														
4.														
5.														
6.														
7.														
8.														
9.														
10														

*** ਸੰਪਰਕ ਦੀ ਕਿਸਮ ਬਾਰੇ ਜਿਕਰ ਕਰੋ**	**ਸੰਖਿਆ**
ਛੂਹਇਆ ਜਾਂ ਚਮੜੀ ਨੂੰ ਚਟਿੱਆ	1
ਨੰਗੀ ਚਮੜੀ ਉਤੇ ਦਵਾਬ, ਛੋਟੀ ਝਰੀਟ ਜਾਂ ਬਿਨਾਂ ਖੁਨ ਵੱਗੇ ਵਾਲਾ ਜਖਮ ਹੋਇਆ	2
ਬਿਨਾਂ ਖੂਨ ਵਗੇ ਜਖਮ ਜਾਂ ਝਰੀਟ ਦਾ ਹਲਕੇ ਹੋਏ ਜਾਨਵਰ ਦੇ ਥੁੱਕ ਦੇ ਸਿੱਧੇ ਸੰਪਰਕ ਵਿੱਚ ਆਓਣ ਨਾਲ	3
ਅੱਖ, ਨੱਕ ਜਾਂ ਮੂੰਹ ਦਾ ਹਲਕੇ ਹੋਏ ਜਾਨਵਰ ਦੇ ਥੁੱਕ ਦੇ ਸਿੱਧੇ ਸੰਪਰਕ ਵਿੱਚ ਆਓਣ ਨਾਲ	4
ਤਾਜੇ ਜਖਮ ਦਾ ਹਲਕੇ ਹੋਏ ਜਾਨਵਰ ਦੇ ਥੁੱਕ ਦੇ ਸਿੱਧੇ ਸੰਪਰਕ ਵਿੱਚ ਆਓਣ ਨਾਲ	5
ਪਸ਼ੂ ਨੂੰ ਛੇੜਣ ਤੋਂ ਬਾਅਦ ਪਸ਼ੂ ਨੇ ਇੱਕ ਜਾਂ ਇੱਕ ਤੋਂ ਵੱਧ ਥਾਂਵਾ ਤੇ ਵੱਡਿਆ ਹੋਵੇ ਤੇ ਜਖਮ ਹੋ ਗਏ ਹੋਣ	6
ਬਿਨ੍ਹਾ ਕੋਈ ਛੇੜਖਾਨੀ ਤੋਂ, ਪਸ਼ੂ ਨੇ ਇੱਕ ਜਾਂ ਇੱਕ ਤੋਂ ਵੱਧ ਥਾਂਵਾ ਤੇ ਵੱਡਿਆ ਹੋਵੇ ਤੇ ਜਖਮ ਹੋ ਗਏ ਹੋਣ	7
ਹਲਕੇ ਹੋਏ ਜਾਨਵਰ ਦੇ ਕੱਚੇ ਦੁੱਧ ਦਾ ਸੇਵਣ ਕਰਨ ਨਾਲ	8

30. ਐਕਸਪੋਜਡ ਮਨੁੱਖ ਦਾ ਨਾਮ ਅਤੇ ਪੱਤਾ?

ਭਾਗ II: ਵੈਟਰਨਰੀ ਡਾਕਟਰ ਅਤੇ ਵੈਟਰਨਰੀ ਫਾਰਮਾਂਸਿਸਟ ਦੇ ਲਈ

ਵਿਅਕਤੀਗਤ ਜਾਣਕਾਰੀ

ਮਾਲਕ ਦਾ ਪਛਾਣ ਕੋਡ(ਜੇ ਕੋਈ ਹੈ): ☐

1. ਨਾਮ ☐

2. ਸਿਵਲ ਵੈਟਰਨਰੀ ਹਸਪਤਾਲ/ ਸਿਵਲ ਵੈਟਰਨਰੀ ਡਿਸਪੈਂਸਰੀ ਦਾ ਨਾਮ ☐

3. ਡਾਕਖਾਨਾ ਕੋਡ ☐

4. ਆਹੁਦਾ? **ਵੈਟਰਨਰੀ ਡਾਕਟਰ** ☐ **ਵੈਟਰਨਰੀ ਫਾਰਮਾਂਸਿਸਟ** ☐

5. ਉਮਰ ______ ਸਾਲ

6. ਲਿੰਗ ਪੁਰਸ਼ ☐ ਇਸਤਰੀ ☐

7. ਕਿੰਨ੍ਹੇ ਸਾਲ ਤੋਂ ਵੈਟਰਨਰੀ ਦਾ ਕੰਮ ਕਰ ਰਹੇ ਹੋ? ☐

8. ਅਪਣੀ ਵੈਟਰਨਰੀ ਪ੍ਰੈਕਟਿਸ ਦੋਰਾਨ ਹਲਕਾ ਦੇ ਕਿਨ੍ਹੇ ਕੇਸ ਦੇਖ ਚੁੱਕੇ ਹੋ? ☐

9. ਕੱਦੇ ਤੁਹਾਨੂੰ ਹਲਕਾ ਦਾ ਐਕਸਪੋਜਰ ਹੋਇਆ ਹੈ? ਹਾਂ ☐ ਨਹੀਂ ☐

ਜੇ ਹਾਂ ਤਾਂ, ਸੰਪਰਕ ਦੀ ਕਿਸਮ ਨੂੰ ਚੁਣੋ*

* ਸੰਪਰਕ ਦੀ ਕਿਸਮ ਬਾਰੇ ਜਿਕਰ ਕਰੋ	ਸੰਖਿਆ
ਛੂਹਇਆ ਜਾਂ ਚਮੜੀ ਨੂੰ ਚਟਿੱਆ	1
ਨੰਗੀ ਚਮੜੀ ਉਤੇ ਦਵਾਬ, ਛੋਟੀ ਝਰੀਟ ਜਾਂ ਬਿਨਾਂ ਖੂਨ ਵੱਗੇ ਵਾਲਾ ਜਖਮ ਹੋਇਆ	2
ਬਿਨਾਂ ਖੂਨ ਵਗੇ ਜਖਮ ਜਾਂ ਝਰੀਟ ਦਾ ਹਲਕੇ ਹੋਏ ਜਾਨਵਰ ਦੇ ਥੁੱਕ ਦੇ ਸਿੱਧੇ ਸੰਪਰਕ ਵਿੱਚ ਆਓਣ ਨਾਲ	3
ਅੱਖ, ਨੱਕ ਜਾਂ ਮੂੰਹ ਦਾ ਹਲਕੇ ਹੋਏ ਜਾਨਵਰ ਦੇ ਥੁੱਕ ਦੇ ਸਿੱਧੇ ਸੰਪਰਕ ਵਿੱਚ ਆਓਣ ਨਾਲ	4
ਤਾਜੇ ਜਖਮ ਦਾ ਹਲਕੇ ਹੋਏ ਜਾਨਵਰ ਦੇ ਥੁੱਕ ਦੇ ਸਿੱਧੇ ਸੰਪਰਕ ਵਿੱਚ ਆਓਣ ਨਾਲ	5
ਪਸ਼ੂ ਨੂੰ ਛੇੜਣ ਤੋਂ ਬਾਅਦ ਪਸ਼ੂ ਨੇ ਇੱਕ ਜਾਂ ਇੱਕ ਤੋਂ ਵੱਧ ਥਾਂਵਾ ਤੇ ਵੱਡਿਆ ਹੋਵੇ ਤੇ ਜਖਮ ਹੋ ਗਏ ਹੋਣ	6
ਬਿਨ੍ਹਾ ਕੋਈ ਛੇੜਖਾਨੀ ਤੋਂ, ਪਸ਼ੂ ਨੇ ਇੱਕ ਜਾਂ ਇੱਕ ਤੋਂ ਵੱਧ ਥਾਂਵਾ ਤੇ ਵੱਡਿਆ ਹੋਵੇ ਤੇ ਜਖਮ ਹੋ ਗਏ ਹੋਣ	7
ਹਲਕੇ ਹੋਏ ਜਾਨਵਰ ਦੇ ਕੱਚੇ ਦੁੱਧ ਦਾ ਸੇਵਣ ਕਰਨ ਨਾਲ	8

ਅ. ਬਿਮਾਰ ਪਸ਼ੂ ਬਾਰੇ ਜਾਣਕਾਰੀ (ਹਲਕਾ ਦੇ ਸ਼ੱਕ ਸੰਬਧੀ)

10. Species of animal ☐

11. Age ______

12. Sex Male ☐ Female ☐

13. ਮੌਤ ਦਾ ਕਾਰਣ ਯੁਥੇਨਾਈਜ਼ ☐ ਹਲਕਾ ਦੌਰਾਨ ਮੌਤ ☐ ਮਾਰਨਾ (ਕਿੱਸ ਤਰ੍ਹਾਂ ਮਾਰਿਆ)? ☐

14. ਬਿਮਾਰੀ ਦੇ ਲਛਣ

☐ ਨਾ ਖਾਣ ਯੋਗ ਵਸਤਾਂ ਨੂੰ ਖਾਣਾ	☐ ਮੂੰਹ ਵਿੱਚੋਂ ਝੱਗ ਆਉਣਾ	☐ ਖੰਘ
☐ ਧੱਸਤ ਆਉਣੇ	☐ ਉਲਟੀ ਆਉਣਾ	☐ ਬੁਖਾਰ
☐ ਲੋਕਾ/ਚੀਜ਼ਾਂ ਤੇ ਦੰਦੀ ਵੱਢਣ ਦੀ ਕੋਸ਼ਿਸ਼ ਕਰਨੀ	☐ ਵਿਅਕਤੀਆਂ ਨੂੰ ਵੱਢਿਆ ਗਿਆ	☐ ਘੁੱਮਣਾ
☐ ਸੁਬਾਹ ਵਿੱਚ ਤੱਬਦੀਲੀ	☐ ਮਾਲਕ ਨੂੰ ਪਛਾਣਨਾ	☐ ਭੁੱਖਾ
☐ ਖੱੜ ਹੋਣ/ਚਲਣ ਵਿੱਚ ਤਕਲੀਫ	☐ ਅਧਰੰਗ	☐ ਖਾਣ ਵਿੱਚ ਤੱਕਲੀਫ

ਕੋਈ ਵੀ ਹੋਰ ਸੰਬੰਧਿਤ ਜਾਣਕਾਰੀ ☐

15. ਕਲੀਨਿਕਲ ਕਿਸਮ

ਗੂੰਗਾ ☐ ਭੜਕਾਓ ☐ ਪੱਤਾ ਨਹੀਂ ☐

16. ਕਲੀਨਿਕਲ ਲਛਣਾ ਦੇ ਅਦਾਰ ਤੇ, ਕੀ ਤੁਸੀ ਸੋਚਦੇ ਹੋ ਇਹ ਇਕ ਹਲਕਾ ਦਾ ਕੇਸ ਹੈ?

ਹਾਂ ☐ ਨਹੀਂ ☐ ਪੱਤਾ ਨਹੀਂ ☐

ਜੇ ਨਹੀਂ ਪਤੱਾ, ਤਾਂ ਤੁਹਾਨੂੰ ਲਗਦਾ ਹੈ ਕਿ ਇਹ ਕੋਈ ਹੋਰ ਬਿਮਾਰੀ ਹੋ ਸਕਦੀ ਹੈ ? ਹਾਂ ☐ ਨਹੀਂ ☐

ਜੇ ਹਾਂ , ਤਾਂ ਇਹ ਕਿਸ ਤਰ੍ਹਾਂ ਦੀ ਬਿਮਾਰੀ ਹੋ ਸਕਦੀ ਹੈ ?

ੲ. ਕੰਮ ਦੇ ਸੰਬਧ ਵਿੱਚ ਐਕਸਪੋਜਰ ਬਾਰ

17. ਕੀ ਤੁਸੀਂ ਹਲਕਾ ਦਾ ਟੀਕਾਕਰਣ ਕਰਵਾਇਆ ਹੋਇਆ ਹੈ? ਹਾਂ ☐ ਨਹੀਂ ☐

18. ਕੀ ਤੁਸੀਂ ਹਰ ਸਾਲ ਹਲਕਾ ਦੇ ਟੀਕੇ ਦੀ ਬੂਸਟਰ ਡੋਜ਼ ਲਗਾਉਂਦੇ ਹੋ? ਹਾਂ ☐ ਨਹੀਂ ☐

19. ਕੀ ਤੁਸੀਂ ਕੱਦੇ ਅਪਣਾ ਐਂਟੀਬਾਡੀ ਟਾਈਟਰ ਦਾ ਚੈਕਅਪ ਕਰਵਾਇਆ ਹੈ? ਹਾਂ ☐ ਨਹੀਂ ☐

20. ਕੀ ਤੁਸੀਂ ਸ਼ੱਕੀ ਹਲਕੇ ਜਾਨਵਰ ਨੂੰ ਦੇਖਣ ਸਮੇਂ, ਨਿੱਜੀ ਸੁੱਰਖਿਆ ਉਪਕਰਨਾਂ ਦੀ ਵਰਤੋਂ ਕਰਦੇ ਹੋ? ਹਾਂ ☐ ਨਹੀਂ ☐

ਜੇ ਹਾਂ, ਤਾਂ ਹੇਠ ਦਿਤੇ ਨਿੱਜੀ ਸੁੱਰਖਿਆ ਉਪਕਰਨਾਂ ਨੂੰ ਚੁਣੋ

ਦਸਤਾਨੇ ☐ ਸਰਜੀਕਲ ਮਾਸਕ ☐ ਬਚਾਅ ਲਈ ਐਨਕਾਂ ☐

ਗਾਊਨ ਜਾਂ ਲੈਬ ਕੋਟ ☐ ਹੋਰ ਉਪਕਰਣ ☐

ਕੋਈ ਵੀ ਨਹੀਂ ☐

21. ਕੀ ਤੁਸੀਂ ਇਸ ਹਲਕੇ ਹੋਏ ਜਾਨਵਰ ਸੰਪਰਕ ਵਿੱਚ ਆਏ ਹੋ? ਹਾਂ ☐ ਨਹੀਂ ☐

ਜੇ ਹਾਂ ਤਾਂ, ਸੰਪਰਕ ਦੀ ਕਿਸਮ ਨੂੰ ਚੁਣੋ*

* ਸੰਪਰਕ ਦੀ ਕਿਸਮ ਬਾਰੇ ਜਿਕਰ ਕਰੋ	ਸੰਖਿਆ
ਛੂਹਇਆ ਜਾਂ ਚਮੜੀ ਨੂੰ ਚਟਿੱਆ	1
ਨੰਗੀ ਚਮੜੀ ਉਤੇ ਦਵਾਬ, ਛੋਟੀ ਝਰੀਟ ਜਾਂ ਬਿਨਾਂ ਖੁਨ ਵੱਗੇ ਵਾਲਾ ਜਖਮ ਹੋਇਆ	2
ਬਿਨਾਂ ਖੂਨ ਵਗੇ ਜਖਮ ਜਾਂ ਝਰੀਟ ਦਾ ਹਲਕੇ ਹੋਏ ਜਾਨਵਰ ਦੇ ਥੁੱਕ ਦੇ ਸਿੱਧੇ ਸੰਪਰਕ ਵਿੱਚ ਆਓਣ ਨਾਲ	3
ਅੱਖ, ਨੱਕ ਜਾਂ ਮੂੰਹ ਦਾ ਹਲਕੇ ਹੋਏ ਜਾਨਵਰ ਦੇ ਥੁੱਕ ਦੇ ਸਿੱਧੇ ਸੰਪਰਕ ਵਿੱਚ ਆਓਣ ਨਾਲ	4
ਤਾਜੇ ਜਖਮ ਦਾ ਹਲਕੇ ਹੋਏ ਜਾਨਵਰ ਦੇ ਥੁੱਕ ਦੇ ਸਿੱਧੇ ਸੰਪਰਕ ਵਿੱਚ ਆਓਣ ਨਾਲ	5
ਪਸ਼ੂ ਨੂੰ ਛੇੜਣ ਤੋਂ ਬਾਅਦ ਪਸ਼ੂ ਨੇ ਇੱਕ ਜਾਂ ਇੱਕ ਤੋਂ ਵੱਧ ਥਾਂਵਾ ਤੇ ਵੱਡਿਆ ਹੋਵੇ ਤੇ ਜਖਮ ਹੋ ਗਏ ਹੋਣ	6
ਬਿਨ੍ਹਾ ਕੋਈ ਛੇੜਖਾਨੀ ਤੋਂ, ਪਸ਼ੂ ਨੇ ਇੱਕ ਜਾਂ ਇੱਕ ਤੋਂ ਵੱਧ ਥਾਂਵਾ ਤੇ ਵੱਡਿਆ ਹੋਵੇ ਤੇ ਜਖਮ ਹੋ ਗਏ ਹੋਣ	7
ਹਲਕੇ ਹੋਏ ਜਾਨਵਰ ਦੇ ਕੱਚੇ ਦੁੱਧ ਦਾ ਸੇਵਣ ਕਰਨ ਨਾਲ	8
